# α-Synuclein Pathology in Synucleinopathies: Mechanisms, Biomarkers, and Therapeutic Challenges

**DOI:** 10.3390/ijms26115405

**Published:** 2025-06-04

**Authors:** Oscar Arias-Carrión, Magdalena Guerra-Crespo, Francisco J. Padilla-Godínez, Luis O. Soto-Rojas, Elías Manjarrez

**Affiliations:** 1 Experimental Neurology, Instituto Nacional de Rehabilitación Luis Guillermo Ibarra Ibarra, Mexico City 14389, Mexico; 2 Tecnologico de Monterrey, Escuela de Medicina y Ciencias de la Salud, Mexico City 14380, Mexico; 3 Laboratory of Regenerative Medicine, Department of Physiology, Faculty of Medicine, National Autonomous University of Mexico, Mexico City 04360, Mexico; mguerra@facmed.unam.mx (M.G.-C.); franciscoj.padilla@iteso.mx (F.J.P.-G.); 4 Department of Mathematics and Physics, Western Institute of Technology and Higher Education, San Pedro Tlaquepaque 45604, Mexico; 5 Laboratory of Molecular Pathogenesis, Building A4, Medical Surgeon Career, Faculty of Higher Studies Iztacala, National Autonomous University of Mexico, Mexico City 54090, Mexico; oskarsoto123@unam.mx; 6 Instituto de Fisiología, Benemérita Universidad Autónoma de Puebla, 14 Sur 6301, Col. San Manuel, Apartado Postal 406, Puebla 72570, Mexico; elias.manjarrez@correo.buap.mx

**Keywords:** alpha-synuclein, synucleinopathies, Parkinson’s disease, biomarkers, neurodegeneration, protein aggregation

## Abstract

Parkinson’s disease and related synucleinopathies, including dementia with Lewy bodies and multiple system atrophy, are characterised by the pathological aggregation of the α-synuclein (aSyn) protein in neuronal and glial cells, leading to cellular dysfunction and neurodegeneration. This review synthesizes knowledge of aSyn biology, including its structure, aggregation mechanisms, cellular interactions, and systemic influences. We highlight the structural diversity of aSyn aggregates, ranging from oligomers to fibrils, their strain-like properties, and their prion-like propagation. While the role of prion-like mechanisms in disease progression remains a topic of ongoing debate, these processes may contribute to the clinical heterogeneity of synucleinopathies. Dysregulation of protein clearance pathways, including chaperone-mediated autophagy and the ubiquitin–proteasome system, exacerbates aSyn accumulation, while post-translational modifications influence its toxicity and aggregation propensity. Emerging evidence suggests that immune responses and alterations in the gut microbiome are key modulators of aSyn pathology, linking peripheral processes—particularly those of intestinal origin—to central neurodegeneration. Advances in biomarker development, such as cerebrospinal fluid assays, post-translationally modified aSyn, and real-time quaking-induced conversion technology, hold promise for early diagnosis and disease monitoring. Furthermore, positron emission tomography imaging and conformation-specific antibodies offer innovative tools for visualising and targeting aSyn pathology in vivo. Despite significant progress, challenges remain in accurately modelling human synucleinopathies, as existing animal and cellular models capture only specific aspects of the disease. This review underscores the need for more reliable aSyn biomarkers to facilitate the development of effective treatments. Achieving this goal requires an interdisciplinary approach integrating genetic, epigenetic, and environmental insights.

## 1. Introduction

Parkinson’s disease (PD), dementia with Lewy bodies (DLB), and multiple system atrophy (MSA) are distinct neurodegenerative syndromes collectively known as synucleinopathies, as they are all marked by the presence of α-synuclein (aSyn) protein within the cytoplasm. These conditions differ based on the specific cellular compartments where aSyn accumulates. In PD and DLB, the protein is primarily found in Lewy bodies (LBs) within neurons, whereas in MSA, it is predominantly observed in glial cytoplasmic inclusions (GCIs) [1,2]. With numbers reflecting a significant public health impact, synucleinopathies have garnered considerable attention in the study of their molecular and pathological processes. Indeed, with approximately 2.5 million people in the United States suffering from some form of synucleinopathy [3] and more than 8.5 million globally [4], the urgency of improving early detection, treatment, and preventative strategies for PD and related synucleinopathies is evident.

aSyn was initially identified through an antibody recognising cholinergic vesicles from the torpedo electric organ [5]. This antibody highlighted aSyn expression at both the presynaptic level and the nuclear envelope, which led to the term “synuclein” [5]. Further studies characterised aSyn as a small protein composed of 140 amino acids. While its precise physiological role remains incompletely understood, it is believed to be involved in neurotransmitter release and facilitates transient synaptic vesicle fusion. The link between aSyn and neurodegeneration was first proposed after its presence was detected in amyloid plaques associated with Alzheimer’s disease [5]. A pivotal breakthrough occurred in 1997 when Polymeropoulos and colleagues identified mutations in the *SNCA* gene in an Italian-American family with early-onset Parkinson’s disease (PD), which was confirmed to exhibit Lewy pathology upon autopsy [6]. That same year, Spillantini and colleagues demonstrated that LBs and Lewy neurites in the substantia nigra of patients with idiopathic PD and DLB exhibited strong aSyn immunoreactivity, firmly classifying PD and DLB as synucleinopathies [7]. A year later, independent research by Spillantini and Wakabayashi revealed the presence of aSyn in the GCIs of MSA patient samples [8,9].

Further key discoveries include findings that, potentially, different aSyn strains preferentially affect specific cell types and regions in the brain [10] and that aSyn aggregation patterns in cerebrospinal fluid differ between PD and MSA [11]. Misfolded aSyn can spread between neurons, forming cytoplasmic and/or nuclear inclusions in adjacent cells [12]. This observation has fuelled suggestions that synucleinopathies share features with prion diseases, although no cases of direct human-to-human transmission have been documented. Notably, the relationship between clinical symptoms and pathological findings is not always straightforward, as aSyn aggregates have been detected in individuals without neurodegenerative disease, as well as in patients with other conditions. Additionally, indirect evidence from human studies suggests that protein aggregation may not always be inherently disease-causing [13].

In this regard, given the significant impact of synucleinopathies in patients, as well as the substantial gaps in our knowledge of aSyn and its physiological and pathological mechanisms, this review explores the physiological structure, aggregation, clearance, and interactions of aSyn, alongside recent advances in biomarker research and mechanistic studies. We aim to highlight the key pathways underlying aSyn pathology and how their disruption contributes to different synucleinopathies. Furthermore, we describe the complexity of synucleinopathies, underscoring the need for reliable biomarkers. Cerebrospinal fluid (CSF) aSyn levels, skin biopsies, and advanced seed amplification assays (SAAs), such as RT-QuiC and PMCA, show promise for early detection. We also highlight animal and cellular models as crucial for studying aSyn pathology, although current models have limitations; new approaches, such as CRISPR/Cas9 and patient-derived iPS cells, offer potential improvements. We finalize by proposing novel potential treatments in line with current investigations.

## 2. Physiological α-Synuclein: A Necessary Disordered Protein

### 2.1. Structure of α-Synuclein

**Figure 1:**
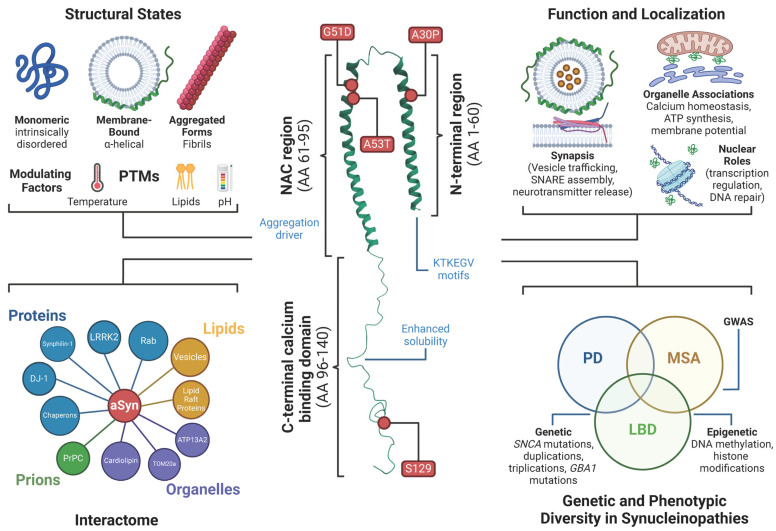
**Structural dynamics, molecular interactions, and pathological roles of alpha-synuclein.*** Structural states of alpha-synuclein.* Alpha-synuclein exists in multiple conformations, including monomeric intrinsically disordered forms, membrane-bound α-helical structures, and aggregated fibrillar states, with its aggregation influenced by post-translational modifications (PTMs), pH, temperature, and lipid interactions. *Molecular interactome.* Alpha-synuclein interacts with a diverse range of molecular partners, including lipid membranes, SNARE proteins, cytoskeletal components, vesicular trafficking machinery, and organelles, modulating its physiological functions and pathological effects. *Physiological functions and localization.* Alpha-synuclein plays a crucial role in synaptic vesicle trafficking, neurotransmitter release, calcium homeostasis, ATP synthesis, and nuclear processes, including transcription regulation and DNA repair. *Genetic and phenotypic diversity in synucleinopathies.* Mutations, duplications, and triplications in *SNCA*, along with environmental and epigenetic factors, contribute to the aggregation and neurotoxicity of alpha-synuclein, underlying the pathogenesis of Parkinson’s disease, multiple system atrophy, and dementia with Lewy bodies.

aSyn is a small, highly dynamic protein composed of three distinct domains that collectively define its structural and functional properties (Figure 1) [14,15]. The N-terminal region contains four imperfect KTKEGV motif repeats, forming amphipathic helices essential for cellular membrane interactions [16]. The central hydrophobic domain, known as the non-amyloid component (NAC), is a key driver of aSyn aggregation and amyloid fibril formation. In contrast, the C-terminal region remains dynamically highly disordered [17,18] and is enriched with acidic and charged amino acids, which enhance solubility and regulate interactions with other proteins and ions [19]. These regions collectively enable aSyn’s functional adaptability in physiological conditions and its pathogenic potential in disease states [20].

Under normal conditions, aSyn is classified as an intrinsically disordered protein (IDP) [21,22], meaning it lacks a stable three-dimensional structure and primarily exists as an unstructured monomer. Its chemical shifts closely resemble a random coil [21,23], suggesting that, like other IDPs [24], its structure may be influenced by temperature and pH. However, upon interaction with cellular membranes or lipids, the N-terminal region adopts an alpha-helical conformation, a structural adaptation that promotes membrane curvature and stability [25,26,27,28]. Consistently, in vitro microfluidic studies that mimic the plasma membrane have shown that aSyn alters membrane topology by inducing pores, increasing membrane capacitance, and immobilizing lipids [29]. Lipids are crucial in modulating aSyn structure and function [25,26,30,31]. Beyond its monomeric and dimeric states, aSyn has been reported to form tetramers and higher-order quaternary structures [32]. However, although studies suggest that these assemblies may be physiologically relevant—potentially regulating neurotransmitter release, glutamatergic receptor activity, and synaptic function [33,34,35,36,37]—their prevalence and functional significance remain debated [17,38].

### 2.2. Function and Subcellular Localization of α-Synuclein

aSyn was initially identified as a protein localized to both the synapse and the nucleus, reflecting its multifaceted functions and complex subcellular distribution (Figure 1) [5]. While its synaptic roles have been extensively studied—primarily due to their connection to neurodegeneration and LB pathology—its roles in the nucleus and other cellular compartments remain relatively underexplored, representing critical gaps in our understanding of its biology [7,39,40]. At the synapse, aSyn exists in a delicate equilibrium between soluble cytosolic and membrane-bound forms. It preferentially binds to lipid rafts enriched with unsaturated and polyunsaturated fatty acids and exhibits a strong affinity for highly curved membranes, such as synaptic vesicles [14,41]. However, the lipid composition modulates aSyn-induced docking of synaptic vesicles on the presynaptic membrane [42]. These properties allow aSyn to modulate essential synaptic processes, including vesicle trafficking, fusion, and neurotransmitter release. Through direct interactions with synaptobrevin, aSyn facilitates SNARE complex assembly, a process critical for vesicle fusion at the presynaptic terminal [43,44].

Beyond the synapse, aSyn is found in several organelles, exerting additional functions. Within mitochondria, aSyn interacts with the inner membrane through cardiolipin and the outer membrane via TOM20, a key protein involved in mitochondrial protein import [45,46]. aSyn also accumulates on the endoplasmic reticulum (ER) and Golgi membranes, where its overexpression disrupts ER–Golgi trafficking, induces ER stress and leads to Golgi fragmentation [47,48]. Additionally, aSyn is associated with mitochondria-associated membranes (MAMs), specialized regions that mediate ER–mitochondria communication and regulate calcium homeostasis. Dysfunction in MAMs has been implicated in synucleinopathies [49]. aSyn interacts with histones and DNA in the nucleus, influencing chromatin acetylation and modulating gene expression and DNA repair processes [50,51]. Although relatively understudied, these nuclear roles suggest broader regulatory functions that may contribute to neurodegenerative pathways.

Outside the nervous system, aSyn is highly expressed in peripheral tissues, particularly within red blood cells, platelets, and the gastrointestinal tract. Over 99% of aSyn in blood is localized to red blood cells, with smaller amounts found in platelets and plasma [52]. The functional roles of aSyn in peripheral tissues are poorly understood [39]. Notably, its detection in the gastrointestinal tract has attracted significant interest, particularly regarding its potential contribution to the gut–brain axis [53,54]. This observation supports the hypothesis that aSyn pathology could originate in peripheral sites and propagate to the central nervous system.

### 2.3. α-Synuclein Interactome Further Complexes Its Understanding

Extensive research has identified various aSyn interacting partners, including proteins, lipids, and nucleic acids. These interactions underscore aSyn’s multifaceted roles in cellular physiology. Notable protein interactors include Synphilin-1 [55], LRRK2 [56,57], p25alpha [58], DJ-1 [59], ATP13A2 [60,61], and chaperones such as heat shock proteins [62,63]. aSyn also engages Rab family proteins to regulate vesicle trafficking [64,65], binds to heparan sulphate proteoglycans, and interacts with nuclear components like histones and DNA, further emphasising its functional versatility [66,67].

In this regard, the expansive aSyn interactome complicates our understanding of its physiological functions. Interactions with lipid membranes further illustrate aSyn’s role in cellular dynamics and disease. As previously described, the N-terminal domain is essential for binding to synaptic vesicles, axonal transport vesicles, and lipid rafts, processes integral to its physiological role in vesicle trafficking [41,68]. However, these interactions can also promote aggregation under pathological conditions. At the presynaptic terminal, aSyn interacts with proteins essential for neurotransmitter release, including Rab3, SNARE proteins, synapsin III, and vesicular monoamine transporter 2 [44,69,70]. Moreover, aSyn regulates dopamine and serotonin transporters, as well as modulates enzymatic activity, such as tyrosine hydroxylase function, further demonstrating its regulatory capacity in synaptic physiology [71,72].

Mitochondrial interactions are another crucial factor in aSyn pathology [73]. Although crucial for maintaining mitochondrial integrity, such interactions may become compromised in disease states, contributing to energy deficits and increased oxidative stress [46,74]. Furthermore, aggregated aSyn exacerbates mitochondrial dysfunction by directly impairing protein import and destabilizing membrane integrity [75]. These impairments amplify cellular stress, increase neuronal vulnerability, and drive the progression of synucleinopathies [76].

However, despite significant progress, many aspects of the aSyn interactome remain poorly defined [77]. Traditional approaches, such as co-immunoprecipitation and crosslinking, have yielded valuable insights but are limited by their specificity and physiological relevance [78]. Emerging methodologies, including mass spectrometry, nuclear magnetic resonance (NMR) spectroscopy, bimolecular fluorescence complementation, and proximity ligation assays, offer improved sensitivity and precision for detecting and characterising aSyn interactions [79]. However, these approaches face challenges, including artefacts from overexpression systems and non-physiological conditions, underscoring the need for rigorous experimental validation [80]. Computational approaches that leverage molecular modelling and protein structure databases have demonstrated promise in predicting aSyn interactions [81]. Nonetheless, these predictions require stringent validation in models that closely mimic the human brain environment [82]. For greater translational relevance, in vitro systems must incorporate physiological expression levels, while in vivo models should recapitulate the tissue-specific contexts of synucleinopathies. High-resolution imaging and proteomics provide novel avenues for elucidating the dynamic nature of aSyn interactions across subcellular compartments [83]. Additionally, functional assays are necessary to distinguish disease-promoting interactions from those that serve protective roles [84].

Deciphering aSyn’s interactome is crucial for understanding its biological functions and identifying potential therapeutic targets [39]. Targeting key interaction nodes within this network may provide strategic intervention points that preserve essential physiological functions while mitigating pathological effects. Integrating advanced experimental methodologies with computational tools holds significant potential for generating a more comprehensive understanding of aSyn’s multifaceted roles in health and disease. These insights could ultimately facilitate the development of targeted therapies for synucleinopathies [39].

### 2.4. Mechanisms of α-Synuclein Clearance

**Figure 2:**
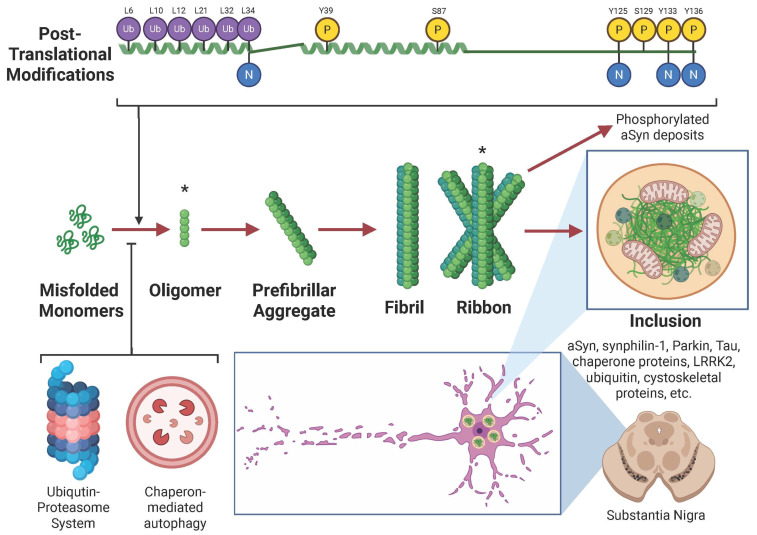
**Alpha-synuclein aggregation, post-translational modifications, and degradation pathways.** Alpha-synuclein misfolds and progressively assembles into soluble oligomers and prefibrillar aggregates, which may evolve into fibrils composed of protofibrillar subdomains *. Post-translational modifications modulate aggregation propensity, while clearance mechanisms such as chaperone-mediated autophagy and the ubiquitin–proteasome system regulate accumulation, with phosphorylation at serine-129 as a key pathological marker. Alpha-synuclein inclusions co-localize with synphilin-1, Parkin, tau, molecular chaperones, LRRK2, ubiquitin, and cytoskeletal proteins, contributing to neurodegeneration. The ubiquitin–proteasome system mediates clearance of alpha-synuclein aggregates and chaperone-mediated autophagy, with dysfunction in these pathways leading to its accumulation, particularly in the substantia nigra.

Given the intrinsically disordered composition of a-Syn and its wide interactome and cellular distribution, the protein is susceptible to becoming pathological (Figure 2). Multiple cellular pathways regulate the aSyn clearance, including chaperone-mediated autophagy (CMA), macroautophagy, and the ubiquitin–proteasome system [85,86]. These mechanisms function to maintain aSyn homeostasis and prevent its pathological accumulation.

Macroautophagy plays a central role in the lysosomal degradation of oligomeric and aggregated aSyn species, as CMA is limited in handling large protein aggregates [87,88]. In contrast, the ubiquitin–proteasome system preferentially targets monomeric aSyn for degradation [89]. Interestingly, the proteasome can also partially process certain soluble oligomeric forms of aSyn [90]. Notably, mutant aSyn variants associated with familial PD, including H50Q, G51D, E46K, and A53E, are preferentially degraded by macroautophagy rather than CMA, suggesting distinct clearance pathways for mutant versus wild-type aSyn [91,92,93].

Beyond intracellular degradation, aSyn is actively secreted into the extracellular environment, where it can be taken up and degraded by neighbouring cells, including glial cells such as astrocytes and microglia, which demonstrate greater capacity for aSyn clearance than neurons [58,94]. However, internalised aSyn seeds can impair the endosomal–lysosomal machinery of recipient cells, promoting the aggregation of endogenous aSyn and propagating pathology [95]. Post-translational modifications (PTMs) further regulate aSyn turnover, influencing its aggregation propensity and degradation efficiency. For instance, ubiquitination, sumoylation, glycation, and phosphorylation modulate aSyn interactions with degradation pathways [96,97,98,99,100]. Their precise role in aSyn degradation remains unclear. Similarly, the effects of other PTMs, such as acetylation and nitration, require further investigation [76]. In this regard, animal models highlight the crucial role of the autophagy–lysosomal pathway in regulating aSyn oligomers and aggregates [101,102,103]. In neurons, lysosomes serve as the primary site for internalised aSyn degradation, while proteases such as calpains [104,105] and metalloproteinases [106] contribute to intracellular and extracellular aSyn clearance [107]. Recent evidence suggests that liquid–liquid phase separation (LLPS) plays a pivotal role in organizing aSyn into biomolecular condensates that may act as nucleation platforms for pathological aggregation. Under physiological and stress conditions, LLPS can concentrate aSyn, promoting transition into amyloid fibrils. This mechanism is particularly relevant in neuronal cells, where crowding and membrane interactions further modulate phase behaviour. However, whether these mechanisms remain efficient during ageing or become impaired in synucleinopathies remains uncertain.

Despite significant advances, key questions regarding aSyn clearance remain unresolved [108]. The relative contributions of different degradation pathways in processing specific aSyn species and the impact of ageing and disease on these pathways require further elucidation. Additionally, the roles of glial cells, particularly microglia and astrocytes, in aSyn clearance are insufficiently characterised despite their likely significance in disease progression [109,110]. Addressing these knowledge gaps requires advanced live-cell imaging techniques to examine the dynamics of aSyn degradation in real time [79]. Tools for modulating and tracking post-translational modifications (PTMs) could provide critical insights into their roles in aSyn clearance [111]. Furthermore, improved reporters and assays for assessing the activity of autophagy, the proteasome, and the lysosomal pathways will facilitate a more comprehensive understanding of their interactions with aSyn [112].

When the pathways responsible for clearing aSyn fail, the protein becomes increasingly vulnerable to misfolding and aggregation. As will be described later, this accumulation of pathological aSyn disrupts cellular homeostasis and contributes to neurotoxicity, ultimately driving the progression of synucleinopathies. A self-perpetuating protein aggregation cycle and neuronal dysfunction occur when these clearance mechanisms fail due to genetic mutations, environmental factors, or ageing-related decline.

## 3. Mechanisms of Aggregation and Pathology

### 3.1. Mechanisms of Aggregation

The dual localization of aSyn as both a cytosolic and membrane-associated protein highlights its physiological versatility while rendering it susceptible to dysregulation. In this regard, synucleinopathies are defined by abnormalities in the normal cellular distribution of aSyn, leading to its accumulation in aggregated forms [12,113,114]. In such conditions, aSyn undergoes dramatic structural transitions, shifting from alpha-helical-rich states to beta-sheet-rich conformations [115]. aSyn misfolds and assembles into a spectrum of aggregates, including soluble oligomers and prefibrillar aggregates, which can evolve into structurally heterogeneous fibrils composed of protofibrillar subdomains (Figure 2) [116,117]. Because toxic aSyn oligomers arise early in aggregation [118], they are considered targets for diagnostic and therapeutic interventions [117]. However, despite extensive research, the molecular mechanisms driving aSyn misfolding remain elusive. Key questions include identifying the factors that initiate aggregation, characterising the toxic structural intermediates, and understanding the cellular conditions that facilitate these pathological changes.

The aggregates derived from aSyn exhibit significant heterogeneity across synucleinopathies, with recent studies identifying distinct conformational structures, such as fibrils and ribbons. It has been proposed that these aggregates represent different “strains” analogous to prion strains and exhibit distinct properties, including variations in size, structure, toxicity, lipid-binding efficacy, and seeding potential [119,120,121]. The diversity of aSyn strains may contribute to the clinical heterogeneity observed in synucleinopathies, as each strain potentially drives unique pathological processes.

Environmental factors, including salt concentration, pH, and bacterial endotoxins, have been shown to influence aSyn aggregation and strain formation [122]. In vivo mouse model studies reveal that different aSyn strains elicit distinct biological effects [123]. Oligomers and ribbons propagate pathology more efficiently than fibrils, yet only ribbons are associated with phosphorylated aSyn deposits, a hallmark of neurodegeneration. Structural polymorphs of aSyn differ in their seeding capacity, toxicity, and propagation dynamics, thereby shaping disease progression and offering potential therapeutic targets [124]. Despite these findings, the strain concept remains unvalidated in human synucleinopathies, and the precise mechanisms driving strain-specific aggregation and toxicity remain elusive.

PTMs add another layer of complexity to regulating aSyn aggregation [125], as described below. Phosphorylation at critical residues is thought to modulate aggregation dynamics, whereas C-terminal truncations—frequently observed in Lewy bodies—are known to enhance aggregation [126]. Additionally, interactions with cellular membranes and lipids play a crucial role, as specific lipid compositions significantly influence aSyn’s propensity to aggregate [127]. However, the mechanisms linking these factors to pathological aggregation remain poorly understood. Intriguingly, recent evidence challenges the conventional view that Lewy pathology is central to diseases like DLB, raising questions about the relative contributions of aSyn aggregates to disease progression [128,129].

A major challenge in studying aSyn aggregation is the variability inherent in experimental systems. Recombinant aSyn, commonly used in aggregation studies, often forms distinct strains under laboratory conditions that may lack physiological relevance, complicating comparisons with human-derived aggregates [130]. This variability underscores the need for standardised aggregation protocols and the careful selection of protein sources to ensure reliable and reproducible findings. Advanced structural techniques, such as cryo-electron microscopy and solid-state nuclear magnetic resonance, are indispensable for elucidating the molecular architecture of aSyn aggregates and bridging the gap between recombinant forms and those extracted from human brain tissue [131,132].

Establishing robust protocols for isolating aggregated aSyn from human brain tissue is a critical research priority [133]. Such protocols are essential for advancing our understanding of the structural and biochemical properties of pathological aggregates and comparing these to experimentally generated forms, thereby enhancing the physiological relevance of in vitro models. Moreover, they must minimise artefacts for meaningful comparisons with recombinant strains and experimental models [134]. If distinct aSyn strains are identified, they could serve as a foundation for conformation-specific therapeutic strategies. Targeting individual strains might enable more precise interventions, although the coexistence of multiple pathological protein species in the brain poses a significant challenge [135]. In particular, mixed pathologies, where aSyn aggregates coexist with other misfolded proteins, such as tau or amyloid-beta, will likely require combinatorial approaches to address each pathological species independently [136,137,138,139,140].

Despite these challenges, substantial progress has been achieved in elucidating the mechanisms underlying aSyn aggregation. Future research should focus on refining experimental models to more accurately replicate the human brain environment, integrating insights from diverse systems, and investigating the functional implications of aSyn strain diversity. Such efforts will advance our understanding of synucleinopathies and inform therapeutic development [141]. This multifaceted approach holds promise for uncovering novel therapeutic targets and advancing our understanding of the complex biology underlying synucleinopathies [39].

One key area of investigation is the mechanistic basis of aSyn pathological propagation. Receptor-mediated internalisation has gained traction as a plausible model for understanding cellular processes. Several receptors, including neurexin 1b, Aβ precursor-like protein 1, and lymphocyte-activating 3, facilitate the uptake of extracellular aSyn, with the latter showing selective binding to aggregated forms [142,143]. Additionally, prion protein (PrPC) has been implicated in sensing toxic α-synuclein oligomers, triggering synaptic dysfunction [142]. These findings support a “prion-like” propagation mechanism, where aggregated aSyn not only oligomerizes but also induces the misfolding of endogenous aSyn, perpetuating its pathological spread across cells.

### 3.2. Role of Post-Translational Modifications and Metals in α-Synuclein Pathology

PTMs and interactions with metal ions have emerged as critical factors in aSyn pathology due to their profound influence on its structure, function, and aggregation dynamics [144,145,146]. The amino acid sequence of aSyn contains distinct sites with varying affinities for metal ions, which modulate its folding, aggregation propensity, and interactions with cellular membranes, contributing to its role in synucleinopathies [147]. Among these, copper (Cu(I/II)) binding at the N-terminal region (1MDVFMK6) and histidine 50 (H50) have been shown to accelerate fibril formation in vitro, even at micromolar concentrations [148,149]. Similarly, calcium (Ca(I/II)) is implicated in aSyn’s role in vesicular transport, with evidence suggesting that Ca(II) and other metals—such as Fe(II), Fe(III), Ni(II), Zn(II), and Mn(II)—preferentially bind to the C-terminal region (119DPNEA125) [150,151]. These metal interactions destabilize monomeric and dimeric aSyn, promoting aggregation and potentially exacerbating disease progression.

PTMs further complicate aSyn pathology by modulating its structural and functional properties [141]. Phosphorylation at serine 129 (S129) is the most extensively studied PTM, though phosphorylation at other sites, such as serine 87 (S87), has also been implicated [152]. Notably, S87 phosphorylation has been shown to reduce fibrillization, suggesting a potential protective role [153,154]. In contrast, phosphorylation of aSyn at Ser129 (pS129) appears to fine-tune neuronal function and activity, reversibly occurring in response to synaptic activity, without direct toxicity [37]. PTMs influence aSyn’s metal-binding properties and aggregation propensity, as seen with phosphorylation at tyrosine 125 (Y125) and S129.

N-terminal acetylation, a physiological PTM, enhances Cu(I) binding, increases aSyn’s alpha-helical content, and improves its membrane-binding capacity [155]. Recent research has also identified lysine acetylation at residues 6 and 10, which mitigates aSyn aggregation and toxicity [156]. Additionally, PTMs such as ubiquitination, nitration, and sumoylation further regulate aSyn’s aggregation dynamics, though their precise roles in synucleinopathies remain poorly understood.

A significant challenge in studying aSyn stems from the reliance on recombinant protein expressed in bacterial systems, which lack eukaryotic-specific PTMs such as N-terminal acetylation [157]. The absence of these modifications raises concerns regarding the physiological relevance of findings derived from non-acetylated aSyn studies, highlighting the need for validation in models that more accurately reflect the native biochemical environment [141].

Critical questions remain regarding PTMs and metal binding in aSyn pathology [158]. For instance, does metal binding to aSyn—particularly Cu and Fe—exacerbate oxidative stress and accelerate neurodegeneration [159]? Moreover, cell-type-specific variations in PTM patterns and their impact on aSyn aggregation and disease propagation remain poorly characterised [128].

Recent findings challenge prevailing models of metal–aSyn interactions, as aSyn purified from blood or brain tissue appears to lack classical metalloprotein properties [160]. This underscores the need for more refined experimental systems and methodologies. Additionally, the potential of PTMs as biomarkers in blood or cerebrospinal fluid (CSF) remains largely underexplored [161]. Significant challenges in assay sensitivity and specificity hinder progress in this area, underscoring the necessity for advancements in detection methodologies.

Current analytical techniques, including mass spectrometry and antibody-based approaches, are limited in their ability to characterise aSyn PTMs comprehensively. Improving these methodologies will be essential to unravel the complex interplay between PTMs and metal interactions [161]. Generating high-quality aSyn variants with specific PTMs will be pivotal for conducting detailed structural and functional analyses.

Future research should prioritise the development of refined model systems that faithfully replicate the cellular environment, allowing for systematic modulation of PTMs and metal interactions [162]. Such advancements will be crucial for elucidating the molecular mechanisms underlying aSyn pathology, ultimately providing a foundation for therapeutic innovation. Deep mutational scanning (DMS) is a powerful technique for mapping sequence–activity relationships through systematic mutagenesis [163]. Recently, DMS studies in yeast have revealed that different structural states of aSyn exhibit differential sensitivities to specific mutations, indicating that this IDP responds to changes in the cellular environment [164]. Additionally, oxidative stress has been shown to influence aSyn metabolism, mainly through mitochondrial damage [165]. Furthermore, advances in structural biology techniques, such as cryo-electron microscopy (cryo-EM), in-cell nuclear magnetic resonance (NMR), and super-resolution microscopy, have provided unprecedented insights into aSyn conformations [166,167,168]. These cutting-edge tools continue to enhance our understanding of aSyn’s structure, localization, and function in both physiological and pathological contexts.

### 3.3. Lewy Bodies and Pathological Inclusions

Although they are significant synucleinopathies, aSyn aggregates are not in the final stage. LBs and LNs are hallmark pathological features of PD and DLB, characterised as eosinophilic inclusions within neuronal cells. First described in 1912 by Fritz Jakob Heinrich Lewy, these structures comprise dense proteinaceous cores surrounded by radially oriented filaments [169,170]. Subsequent studies revealed that LBs are enriched with ubiquitin [171,172] and aSyn, their principal protein component [7]. Notably, phosphorylated aSyn at serine 129 and other PTMs are consistently detected within LBs, highlighting its critical role in synucleinopathy pathogenesis [173].

The molecular complexity of LBs suggests a multifaceted role in disease pathology. In addition to aSyn, over 90 proteins have been identified within LBs, including synphilin-1 [174], Parkin [175], tau [176], chaperone proteins [63], and leucine-rich repeat kinase 2 (LRRK2) [177]. These proteins encompass structural and binding elements, components of the ubiquitin–proteasome system, and factors involved in cellular stress responses, cytoskeletal integrity, cell cycle regulation, and intracellular signalling pathways [178]. Advanced imaging techniques have revealed the presence of membranous components, including mitochondria and lysosomes, within LBs. Interestingly, these studies suggest a relatively low abundance of aSyn fibrils, indicating a more complex assembly process than previously understood [129]. This biochemical heterogeneity highlights the need for further investigation into the mechanisms governing LB formation and their biological significance in synucleinopathies.

The anatomical distribution of LBs aligns with the clinical symptoms observed in PD and DLB [179]. LBs are predominantly located in dopaminergic neurons within the substantia nigra (SN), contributing to the motor deficits characteristic of PD [180,181]. Additionally, they are present in glutamatergic pyramidal neurons of the limbic system and neocortex and cholinergic neurons in the basal forebrain, correlating with cognitive and behavioural symptoms [182,183,184]. This selective vulnerability highlights the complex interplay between LB pathology and neuronal populations.

The functional role of LBs remains an area of ongoing debate. Studies diverge on whether LBs exert toxic, inert, or protective effects. Some evidence suggests a correlation between LB burden and symptom severity [185,186,187], whereas other findings indicate that neurons harbouring LBs may exhibit resilience against degeneration [188]. Several hypotheses have been proposed to reconcile these discrepancies, including (a) the rapid death of neurons prone to LB formation, leaving behind LB-resistant neurons; (b) subpopulations of LBs with varying toxicities; and (c) the possibility that LB formation is a neuroprotective response to toxic intermediates. Notably, familial PD cases that lack LB pathology provide support for the latter hypothesis [189].

A combination of biochemical and functional factors likely contributes to LB formation [190]. Neurons with high metabolic demands, calcium dysregulation, and reliance on protein degradation pathways may be predisposed to LB accumulation [191]. Furthermore, interactions with other neuropathological hallmarks, such as tau aggregation, add further complexity to the picture [192]. Although peripheral aSyn aggregation has been observed, the clinical significance of these findings remains unclear.

Advancing our understanding of LBs requires high-resolution studies of their biochemical composition and molecular architecture [193]. Techniques such as mass spectrometry and selective antibodies are instrumental in identifying key components and their interactions [194]. Elucidating the modulators of LB formation could reveal new therapeutic targets [190]. Access to high-quality post-mortem brain tissues from well-maintained brain banks remains essential for these investigations.

Developing aSyn-specific ligands for PET imaging offers a promising tool for tracking LB pathology in vivo [195,196]. Such imaging modalities could provide crucial insights into the spatial and temporal dynamics of LB progression. In parallel, patient-derived iPS cells and three-dimensional cell culture models serve as valuable platforms for recapitulating LB formation and evaluating therapeutic interventions [197]. However, further validation is required to enhance the reliability of these models.

By integrating advanced biochemical, imaging, and model-based approaches, researchers can unravel the complexities of LBs, paving the way for targeted therapies and improved diagnostic tools.

### 3.4. Genetic and Phenotypic Diversity in Synucleinopathies

The three primary synucleinopathies—PD, DLB, and MSA—are characterised by overlapping yet distinct clinical and pathological features [198]. Despite well-documented clinical distinctions, the mechanisms driving the phenotypic diversity among these diseases remain poorly understood [199]. Genetic and environmental factors and ageing play significant roles in modulating their pathogenesis, but the interplay between these elements remains a key area of investigation [200].

Although most synucleinopathy cases are sporadic, genetic studies have illuminated their molecular underpinnings [34,201]. Early research identified mutations in the *SNCA* gene, which encodes aSyn, as the first genetic link to familial PD [167,202]. Mutations, such as A53T, E46K, and A30P, along with rare *SNCA* locus multiplications, directly increase aSyn levels or alter its aggregation properties, thereby promoting pathology [202]. However, it remains unclear why these mutations predispose individuals to specific synucleinopathies, such as PD versus DLB or MSA.

Genome-wide association studies (GWASs) have expanded our understanding of genetic contributions by identifying variants associated with PD [203], DLB [204], and MSA [205]. These findings underscore the complexity of genetic risk, as these diseases involve multiple low-penetrance variants rather than single high-penetrance mutations. For example, Leucine-rich repeat kinase 2 (*LRRK2*) and *Parkin RBR E3 ubiquitin–protein ligase* (*PARK2*) mutations contribute to the PD spectrum but exhibit phenotypic variability [34,201]. *LRRK2* variants, the most common genetic cause of familial PD, are not always accompanied by Lewy pathology, as evidenced by post-mortem analyses showing pure nigral degeneration in some carriers [206]. Similarly, *PARK2* mutations, commonly linked to early-onset parkinsonism, are often observed in cases lacking Lewy body pathology [6]. These observations highlight the heterogeneity of synucleinopathies and underscore the need for more in-depth investigations into genotype–phenotype correlations to better understand disease mechanisms.

Another key genetic link involves mutations in the *Glucocerebrosidase Beta 1 (GBA1) gene, which encodes glucocerebrosidase, an enzyme critical for lysosomal function* [207]. Heterozygous *GBA1* mutations significantly increase the risk of developing PD and DLB and may contribute to MSA [208]. The association between glucocerebrosidase dysfunction and aSyn pathology suggests that lysosomal impairments and autophagy defects play pivotal roles in synucleinopathies [209,210]. This connection underscores the broader role of lysosomal biology in regulating aSyn homeostasis and pathology.

Genetic studies of synucleinopathies reveal both shared and disease-specific mechanisms [211]. For instance, the role of aSyn aggregation in PD, DLB, and MSA suggests overlapping pathogenic pathways, while disease-specific genetic factors may dictate phenotypic expression [212]. Interestingly, duplication or triplication of the *SNCA* gene is associated with early-onset parkinsonism and atypical symptoms, including hallucinations, cognitive impairment, and dementia [213,214]. However, the rarity of *SNCA* mutations highlights the influence of additional factors, such as epigenetic modifications, environmental exposures, and cellular context, in modulating disease phenotypes [215].

Epigenetic mechanisms, such as DNA methylation and histone modifications, are emerging as critical regulators of synucleinopathies [216,217]. Epigenetic modifications may regulate aSyn expression, modulate neuronal vulnerability, and shape immune responses, shedding light on the heterogeneity of these diseases [216]. The plasticity of the epigenome presents a promising opportunity to identify modifiable risk factors and guide the development of personalised therapeutic strategies.

Integrating genetic findings with neuropathological and mechanistic studies is crucial for advancing our understanding of synucleinopathies [167]. Patient-derived materials, such as iPS cells, allow researchers to study disease-relevant genetic variants in their appropriate cellular contexts [218]. Additionally, improved in vivo models incorporating multiple genetic and environmental factors are needed to recapitulate the complexity of human synucleinopathies [123]. Furthermore, defining subtypes of synucleinopathies through genetic and epigenetic insights holds significant potential for advancing personalised medicine [219]. For example, patients harbouring *GBA1* mutations may benefit from therapies targeting lysosomal dysfunction, whereas those with *LRRK2* variants could respond favourably to kinase inhibitors [220]. This stratified approach can potentially enhance therapeutic efficacy while reducing the variability in treatment outcomes.

The emerging field of multi-omics, which integrates genomics, transcriptomics, proteomics, and metabolomics, holds promise for uncovering previously unrecognised pathways underlying synucleinopathies [221]. Coupling these approaches with advanced computational tools enables researchers to identify novel biomarkers and therapeutic targets, providing new insights and addressing critical knowledge gaps in the field.

## 4. Propagation and Spreading

### 4.1. The Role of α-Synuclein Spreading in Pathology

The biological relevance of aSyn spreading has garnered increasing support, with evidence suggesting that it is more than a secondary phenomenon. Notably, Lewy pathology has been detected in grafted neurons of PD patients, indicating intra-brain transmission of aSyn pathology [118,222]. Animal models further corroborate the ability of misfolded aSyn to propagate across neural circuits. However, confirmation of aSyn transmission at endogenous levels in human synucleinopathies remains elusive [223].

**Figure 3:**
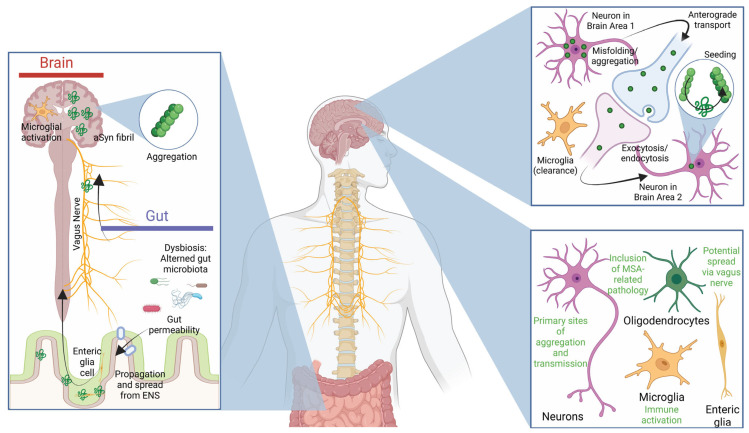
**Propagation and cellular interactions of alpha-synuclein pathology.** Alpha-synuclein aggregates propagate between neurons through anterograde transport, exocytosis, endocytosis, and seeding mechanisms, thereby contributing to the spread of disease across brain regions. Microglia play a crucial role in alpha-synuclein clearance, but their activation may also exacerbate neuroinflammation. In multiple system atrophy (MSA), oligodendrocytes harbour alpha-synuclein inclusions, further driving disease pathology. The gut–brain axis is implicated in early alpha-synuclein aggregation, with enteric glial cells, increased gut permeability, and dysbiosis influencing neurodegenerative processes. Transmission via the vagus nerve has been proposed as a route for gut-derived alpha-synuclein pathology to reach the central nervous system, highlighting a potential mechanism for disease initiation and progression.

The propagation of aSyn pathology across interconnected brain regions is hypothesised to occur by converting physiological aSyn into aggregated or pathological forms (Figure 3) [224,225]. This spreading is proposed to occur in a “prion-like” manner, where misfolded aSyn serves as a template for the conformational transformation of native aSyn. However, this concept remains debated, with unresolved questions about its physiological relevance and pathological mechanisms. Several pathways for aSyn transmission have been proposed, including direct diffusion across cell membranes, secretion via extracellular vesicles, and intercellular transfer through tunnelling nanotubes [226]. Additionally, aSyn fibrils can interact with heparan sulphate proteoglycan chains on the plasma membrane, facilitating macropinocytosis as a mechanism for cellular uptake [227]. These processes exhibit cell-type specificity, predominantly in neurons and oligodendrocytes [66,228].

The extent to which aSyn pathology disseminates among different cell types, including neurons and glia, is not yet fully understood [39,229]. Microglia have been implicated in the uptake and processing of aSyn fibrils through phagocytosis, followed by lysosomal cleavage mediated by asparagine endopeptidase (AEP), potentially contributing to the propagation of pathology [230]. Furthermore, whether aSyn spreads from peripheral tissues, such as the gut or olfactory bulb, to the brain—or vice versa—remains unproven in humans [53,54,231]. Current insights into these processes primarily derive from animal models, which may not fully replicate human synucleinopathies.

A critical question in the field is whether aSyn spreading represents a physiological process or a pathological epiphenomenon [223]. The involvement of PTMs in modulating aSyn’s aggregation and transmissibility is an area of active investigation [232]. For example, phosphorylation at S129 and truncations in the C-terminal region may enhance aSyn’s seeding potential, yet their precise roles remain unclear [233]. Moreover, whether synucleinopathies should be classified as “prion” disorders or primarily as protein aggregation diseases remains a topic of debate [234,235].

Addressing these knowledge gaps requires advanced cellular and animal models that accurately reflect endogenous aSyn expression levels and the cellular complexity of the human brain [141,236,237]. Such models should mimic the endogenous aSyn expression levels and the cellular diversity of the human brain. Investigating how aSyn is released, internalised, and trafficked between cells, as well as how post-translational modifications (PTMs) influence these processes, could identify critical intervention points [111]. High-resolution imaging and proteomic approaches will be crucial for visualising the dynamics of aSyn spreading and mapping its molecular interactions.

Therapeutic strategies targeting aSyn spreading must carefully balance its potential dual role in physiology and pathology. While blocking intercellular transmission may mitigate disease progression, it could also interfere with normal aSyn function [39]. Thus, selectively targeting pathological aSyn conformations while preserving its normal function is crucial. Advancements in experimental models and innovative technologies will be instrumental in refining our understanding of aSyn transmission and developing effective therapeutic interventions for synucleinopathies.

### 4.2. Immune Responses and the Microbiome in Parkinson’s Disease Pathogenesis

Post-mortem studies of PD brains consistently reveal pronounced microgliosis in regions affected by aSyn pathology, including the SN, hippocampus, and cortex [238,239,240,241]. These findings suggest that microglial activation is not merely a consequence of neuronal loss but represents an active and potentially maladaptive response to aSyn pathology. Neurons release aSyn, especially in aggregated forms, which elicit immune responses by engaging microglia and macrophages to clear extracellular aSyn [96,242]. However, the efficiency of these immune cells depends on the aggregation state of aSyn, with misfolded species inducing stronger and potentially detrimental inflammatory responses [243], in part through activation of the NLRP3 inflammasome in microglia [244,245].

Microglia play a dual role in the pathogenesis of PD. On the one hand, they may protect neurons by clearing toxic aggregates of aSyn; on the other, chronic microglial activation exacerbates neuroinflammation, amplifying pro-inflammatory signals and promoting neurodegeneration [96,242,246]. This delicate balance between neuroprotection and neurotoxicity underscores the complexity of microglial responses in PD. Persistent inflammation may establish a self-perpetuating cycle, wherein the immune response to aSyn pathology accelerates neuronal damage, leading to further aSyn release and sustained microglial activation [247]. This pro-inflammatory milieu, driven by pathological aSyn aggregates, can facilitate peripheral immune cell infiltration through chemotaxis or BBB disruption, further exacerbating neuroinflammation [248].

Emerging evidence suggests that the gut microbiome plays a critical role in PD pathogenesis. Studies in PD patients consistently report alterations in gut microbial composition, including reduced bacterial diversity and signs of gastrointestinal inflammation [249,250,251]. The PD-associated microbiota is characterised by increased *Akkermansia* and *Bifidobacterium* and decreased *Roseburia* and *Faecalibacterium*. Furthermore, the depletion of butyrate-producing bacteria and excessive mucus degradation by *Akkermansia* may contribute to intestinal inflammation and increased gut permeability, facilitating the translocation of harmful metabolites into the enteric nervous system (ENS) [252]. Dysbiosis-induced inflammation may drive aSyn accumulation in the gut, promoting its propagation to the brain via the gut–brain axis and triggering neuroinflammation, dopaminergic neuron loss, and ultimately PD progression [253]. Moreover, gut microbiota alterations may serve as biomarkers and disease modifiers, as microbiome imbalances can induce aSyn aggregation in animal models, highlighting a possible causal link [254] and the potential for implementing microbiome-based therapies [255].

The hypothesis that aSyn pathology originates in peripheral tissues, such as the gut or nasal epithelia, before spreading to the CNS has gained increasing support [256,257,258]. Phosphorylated aSyn aggregates have been identified in the ENS of PD patients [259,260], and experimental models have demonstrated that exogenous aSyn fibrils can propagate from the gut to the brain via the vagus nerve [261,262]. Afferent fibres of the vagus nerve are capable of sensing microbiota-derived metabolites, such as lipopolysaccharide (LPS) and short-chain fatty acids (SCFAs), and transmitting these signals to the brainstem via the nucleus tractus solitarii, a component of the dorsal vagal complex [263]. This pathway may contribute to increased susceptibility to PD [264].

Epidemiological studies further suggest that surgical interventions, such as appendectomy and vagotomy, are associated with a reduced risk of PD [265,266,267,268]. Despite these advances, significant gaps remain in understanding the interplay between the gut, the immune system, and aSyn pathology [111]. Key outstanding questions include whether peripheral immune cells encounter aSyn aggregates before microglial activation in the CNS, the mechanisms underlying peripheral-to-central communication during disease progression, and the role of post-translational modifications in modulating immune responses.

Additionally, the direct interaction between microglia and neurons warrants further investigation. The discovery of Lewy body-like structures, including phosphorylated S129-positive inclusions, within microglial cells suggests a direct pathological link between neurons and microglia [233]. However, the consequences of these inclusions for microglial function remain unknown. Do microglial cells with aSyn inclusions remain viable and functional, or do they succumb to cell death? Additionally, why do aSyn inclusions exhibit distinct characteristics across cell types, such as microglia and oligodendrocytes in multiple system atrophy (MSA)?

Addressing these questions requires advanced models and methodologies. Patient-derived organoids, in vivo imaging, and molecular profiling technologies will be crucial for characterising immune responses in both experimental and clinical contexts [269]. These approaches could reveal the dynamic interactions between neuroinflammation and the gut–brain axis in the pathogenesis of PD, providing key insights for the development of diagnostic and therapeutic strategies. Identifying precise modulators of inflammation and aSyn pathology, whether through microbial interventions, anti-inflammatory strategies, or targeted therapies, represents a promising avenue for mitigating disease progression in synucleinopathies [270].

## 5. Models of Synucleinopathies

### 5.1. Animal Models of Synucleinopathies Through α-Synuclein Expression

The development of animal models for studying synucleinopathies has been pivotal in advancing our understanding of aSyn aggregation, neuronal dysfunction, and neurodegeneration [34,151]. These models, primarily based on the expression of wild-type or mutant aSyn, successfully replicate key features of PD and related disorders. However, while early expectations envisioned comprehensive models fully mirroring human synucleinopathies, most only recapitulate specific facets of αSyn biology [271]. Despite these limitations, they remain invaluable tools for dissecting molecular pathways and evaluating therapeutic interventions.

A wide range of transgenic models has been created, including worms (*C. elegans*), flies (*D. melanogaster*), mice, and rats, each offering unique advantages [272]. Invertebrate models facilitate high-throughput genetic and pharmacological screening, bridging the gap between cell-based and whole-organism studies [273,274,275,276]. Mammalian models, particularly transgenic mice, are commonly employed to investigate tissue-specific aSyn expression using neuron-specific promoters [277]. While these models have significantly enhanced our understanding of α-synuclein aggregation and neurodegeneration, they often fail to replicate human disease pathology fully.

Viral vector-mediated models utilizing adeno-associated viruses (AAVs) or lentiviruses offer precise spatiotemporal control of aSyn overexpression via stereotactic brain injections [278,279]. These models facilitate targeted investigations of specific brain regions and neural circuits, enhancing their translational relevance. Additionally, pre-formed fibrils (PFFs) of aSyn represent a significant advancement, as their injection into the brain induces the aggregation of endogenous aSyn, providing a robust platform for studying the propagation and pathogenic mechanisms of aSyn pathology [280,281]. PFF models effectively mimic the prion-like spreading of αSyn pathology, a hallmark of disease progression in PD and related synucleinopathies [282,283], thus allowing interventional studies, such as neuromodulation with optogenetics [284] or engineered binding proteins intended to neutralize aSyn [285]. Notably, PFF inoculation in the duodenal wall of mice led to gastrointestinal deficits and enteric nervous system alterations, reinforcing the role of peripheral synucleinopathy in early PD. Furthermore, aged mice, but not younger ones, exhibited midbrain pathology and motor deficits following αSyn fibril inoculation, highlighting age-dependent susceptibility to neurodegeneration [286].

Despite significant technological advancements, existing models face notable limitations. While many transgenic models successfully induce aSyn aggregation, the resulting inclusions often fail to replicate the ultrastructural and biochemical characteristics of human LBs [287]. The reasons for this discrepancy remain unclear and warrant further investigation. Similarly, consistent neurodegeneration, particularly of dopaminergic neurons in the substantia nigra, is rarely observed, leaving critical questions about selective neuronal vulnerability unresolved [288]. Addressing these gaps is essential for developing more accurate and predictive models that better reflect the complexity of human synucleinopathies.

Several neurotoxin-induced models replicate specific features of PD, but they often fail to capture the slow, progressive, and irreversible neurodegeneration and do not induce αSyn pathology [289]. Recently, a murine model using intranigral administration of the neurotoxin BSSG was developed, successfully mimicking many hallmark features of PD, including motor and non-motor symptoms, nigrostriatal neurodegeneration, neuroinflammation, oxidative stress, and pathological aSyn aggregation and propagation. This model presents a promising platform for studying disease mechanisms and testing therapeutic interventions [290,291].

Emerging technologies, such as CRISPR/Cas9 genome editing, promise to create refined models of PD and synucleinopathies. These tools allow precise genetic modifications to introduce disease-relevant mutations, replicate human-like expression profiles, and manipulate regulatory elements [292]. Incorporating post-translational modifications (PTMs) and protein interactors into these models could enhance their physiological relevance. Insights from tauopathy models emphasise the need to minimise artefacts and rigorously characterise models to ensure reproducibility and reliability [293], setting a benchmark for advancing aSyn research. Knock-in mouse models are valuable for studying neuroinflammation, neurodegeneration, and αSyn-mediated gastrointestinal and olfactory dysfunctions [294,295].

Expanding model development to non-human primates presents a promising avenue for synucleinopathy research. Knock-in primate models can better represent human brain circuits and physiological mechanisms, enhancing translational potential [296]. These models could serve as more accurate platforms for testing therapeutic strategies, helping to bridge the translational gap between preclinical findings and clinical applications.

Future research should focus on developing models that comprehensively integrate key pathological features, including aSyn aggregation, Lewy body formation, neurodegeneration, and cell-type-specific vulnerabilities [272]. Leveraging advanced genetic tools alongside high-resolution imaging, proteomics, and electrophysiology will provide deeper insights into the multifaceted roles of aSyn in health and disease. These integrated approaches are essential for identifying novel therapeutic targets and accelerating the development of disease-modifying treatments for synucleinopathies.

### 5.2. Cellular Models of α-Synuclein Toxicity and Aggregation

Cellular models have been pivotal in elucidating the biology, aggregation dynamics, and toxicity of aSyn. These models span a broad spectrum, including yeast, neuronal and non-neuronal mammalian cell lines, primary neuronal cultures, and patient-derived iPS cells, offering a simplified yet versatile approach to studying aSyn pathology [287,297]. Transient or stable expression systems facilitate the investigation of wild-type and PD-associated mutant forms of aSyn, enabling cost-effective and ethically viable research avenues [298,299]. However, their ability to fully recapitulate the complexity of human synucleinopathies remains limited.

To induce aSyn aggregation, cellular models often rely on exogenous stressors such as 1-methyl-4-phenylpyridinium, rotenone, paraquat, or proteasome inhibitors [300], as overexpression alone frequently fails to form inclusions [301,302]. A notable advancement involves adding extracellular PFFs to primary neuronal cultures, which triggers the recruitment of endogenous aSyn into ubiquitinated and hyperphosphorylated aggregates resembling the pathological hallmarks of synucleinopathies [303]. These models have significantly contributed to uncovering links between aSyn and mitochondrial dysfunction, oxidative stress, and impaired protein degradation pathways [304,305,306]. Furthermore, cellular systems have facilitated the identification of surface proteins that interact with aSyn [142,143] and supported studies on glial degeneration in MSA, mainly through the coexpression of aSyn and p25α in oligodendroglial cell lines [307].

Despite these contributions, several limitations persist. The precise mechanisms underlying aSyn aggregation and cytotoxicity remain incompletely understood. Soluble aSyn oligomers are hypothesised to mediate cellular dysfunction and death during the early stages of aggregation; however, their exact roles remain elusive [306,308,309]. In MSA, accumulating aSyn in oligodendrocytes presents unresolved questions regarding its origin and pathogenic mechanisms [310]. Moreover, proteins such as synphilin-1 [55,311] and p25α [58,312] have been implicated in modulating aSyn aggregation. However, their precise roles remain unclear, suggesting the likely existence of additional, yet unidentified, regulatory factors that influence the aggregation process.

Methodological constraints further challenge the field. The widespread use of antibodies and fluorescent tags for detecting aSyn aggregation may inadvertently alter its biological properties, potentially introducing experimental biases. Addressing these issues requires the development of more specific antibodies and non-invasive tagging strategies to enable precise characterisation of aSyn species in physiologically relevant contexts. Moreover, current cell models produce LB-like inclusions rather than proper LBs, emphasising the need for refined approaches to better mimic disease pathology [190,306,313]. Although iPS cell-derived models hold promise, epigenetic alterations introduced during reprogramming may compromise their fidelity in replicating disease phenotypes.

Future efforts should focus on developing models that integrate genetic, epigenetic, and environmental factors contributing to synucleinopathies. Advanced technologies, such as CRISPR/Cas9 for precise genetic editing and organoid-based 3D cultures, can bridge the gap between traditional cellular models and in vivo systems [314]. Refining existing tools and incorporating novel approaches will enable the creation of more physiologically relevant models, enhancing our understanding of aSyn pathology and accelerating the discovery of targeted therapeutic strategies.

## 6. Towards Diagnostic and Therapeutic Strategies

### 6.1. Biomarkers for Diagnosis

The diagnosis of PD primarily relies on the clinical evaluation of motor symptoms, which typically manifest at advanced disease stages [315]. These motor features often overlap with those of other synucleinopathies, further complicating early diagnosis. Although genetic testing offers valuable insights into familial cases, most PD cases are sporadic, limiting its widespread applicability [316]. These challenges underscore the urgent need for reliable biomarkers to facilitate early detection, differentiate PD from related disorders, and effectively monitor disease progression.

**Figure 4:**
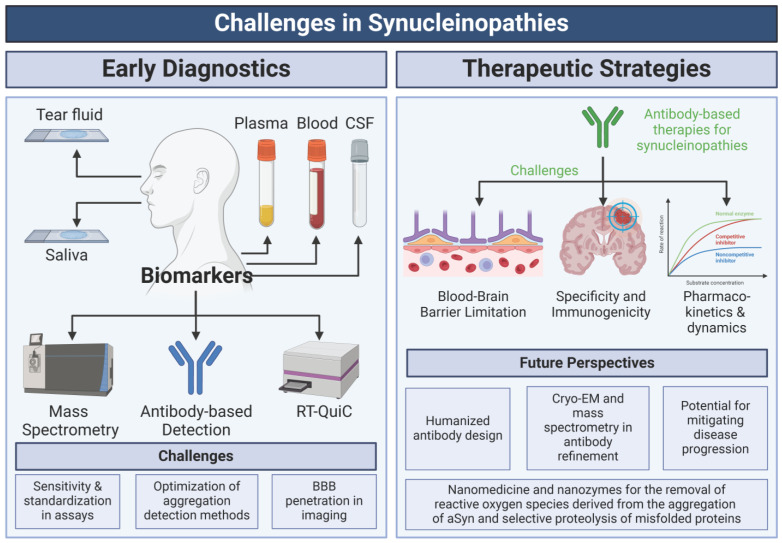
**Challenges and future perspectives in synucleinopathy diagnostics and therapeutics.** Early diagnosis of synucleinopathies relies on the detection of alpha-synuclein biomarkers in cerebrospinal fluid, saliva, plasma, and tear fluid using techniques such as mass spectrometry, antibody-based detection, and real-time quaking-induced conversion (RT-QuIC). However, challenges remain in assay sensitivity, standardisation, and optimising methods for aggregation detection. Imaging approaches are further limited by blood–brain barrier penetration. Therapeutic strategies, including antibody-based therapies, face hurdles related to specificity, immunogenicity, pharmacokinetics, and pharmacodynamics. Future perspectives involve the development of humanized antibodies, advanced cryo-electron microscopy (cryo-EM) and mass spectrometry techniques for antibody refinement, and the use of nanomedicine and nanozymes to target reactive oxygen species and selectively degrade misfolded alpha-synuclein aggregates, offering potential avenues for disease modification.

aSyn has emerged as a promising biomarker candidate (Figure 4). Detectable in CSF, plasma, blood, saliva, and even tear fluid, aSyn and its post-translationally modified forms have been extensively studied for their diagnostic potential [317,318,319]. Among these, CSF-based assays have shown the most significant promise, with studies suggesting that CSF’s ratio of aSyn isoforms could serve as a sensitive and specific diagnostic tool [320,321,322]. PTMs of aSyn, including phosphorylation and truncation, have also been implicated as potential biomarkers measurable in CSF and blood [99,323].

Despite significant advancements, several challenges remain. A critical hurdle is the development of sensitive and standardised assays to quantify aSyn and PTMs across diverse biological fluids accurately. Cutting-edge methodologies, such as advanced mass spectrometry and optimised antibody-based detection techniques, ensure reproducibility and inter-laboratory consistency [324]. These assays must account for variations in aSyn levels across sample types, disease stages, and patient populations to enhance their diagnostic and prognostic utility.

The second major challenge lies in advancing PET imaging for aSyn. Developing PET tracers capable of detecting various forms of aSyn would enable non-invasive, longitudinal studies in patients and individuals at risk. However, selectivity and BBB penetration remain significant obstacles to the practical application of imaging [325,326]. Overcoming these limitations could revolutionize the in vivo tracking of aSyn pathology, providing insights into disease progression and therapeutic responses.

A third area of focus involves leveraging innovative aggregation detection techniques. Methods such as real-time RT-QuiC and protein misfolding cyclic amplification have shown high sensitivity in detecting aggregated, pathology-associated aSyn. These approaches rely on the amyloid-specific interactions of aSyn fibrils with thioflavin T, producing characteristic emission spectra. While RT-QuiC has demonstrated promising results in detecting PD using CSF samples, further optimisation is required to enhance its reliability for widespread clinical use [327,328].

Integrating these tools into clinical and research settings is essential for advancing PD diagnostics. Establishing standardised protocols for aSyn measurement across various fluids and disease stages will improve the accuracy of disease burden and progression assessments. Moreover, developing these biomarkers offers the potential for early intervention, improved differentiation of PD from related disorders, and precise monitoring of therapeutic efficacy.

Addressing these challenges will require a multidisciplinary effort to refine detection technologies, optimise imaging methodologies, and enhance experimental reproducibility. Longitudinal studies employing these refined biomarkers can potentially deepen our understanding of PD pathophysiology, ultimately paving the way for more targeted and effective therapeutic strategies that improve patient outcomes.

### 6.2. Therapeutic Strategies

Antibodies are essential tools for both basic and clinical research, widely employed in immunostaining, immunoblotting, immunoprecipitation, and diagnostics. Recently, their therapeutic potential has been explored for neurodegenerative diseases, including PD and related synucleinopathies [329,330]. With their high specificity, strong antigen-binding properties, and extended half-life, antibodies are promising candidates for immunotherapy. Notably, conformation-specific antibodies targeting aggregated forms of aSyn have shown efficacy in cell and animal models, with several progressing through clinical trials [331,332,333]. However, despite their potential, antibody-based therapies face significant challenges that must be overcome to realize their full therapeutic potential. One major limitation is their restricted ability to cross the BBB, as their large molecular size hinders access to intracellular and CNS targets. Additionally, antibodies with low homology to human proteins may provoke immune responses, leading to neutralization and reduced efficacy. To address this, ongoing efforts focus on developing humanized antibodies to minimise immunogenicity and enhance therapeutic safety.

Another critical challenge is identifying the optimal aSyn conformation to target. While aggregated forms such as oligomers and fibrils are pathological hallmarks of synucleinopathies, their precise roles in disease progression remain controversial. Conformation-specific antibodies have shown promise; however, a deeper understanding of the molecular mechanisms driving aSyn pathology is essential to prioritise therapeutic targets. For instance, while most therapeutic antibodies in clinical trials target the C-terminal region of aSyn, truncation of this region has been implicated in promoting aggregation [334]. Furthermore, PTMs that modulate aSyn structure and aggregation propensity must be carefully considered to enhance antibody specificity and therapeutic efficacy.

Pharmacokinetics and pharmacodynamics pose additional challenges in developing antibody-based therapies for synucleinopathies. Key factors must be optimised to maximise therapeutic efficacy, including antibody binding affinity, avidity, cellular internalisation, and stability. One promising approach involves developing smaller antibody fragments, such as single-chain variable fragments (scFvs) [335], which exhibit improved BBB permeability and intracellular delivery, thereby increasing the therapeutic potential [329,336]. Additionally, therapeutic antibodies must selectively neutralize pathological aSyn species while preserving the physiological functions of aSyn to minimise off-target effects and ensure safety.

Robust preclinical and clinical evaluations are essential for refining antibody design and establishing therapeutic efficacy against synucleinopathies. Longitudinal studies are particularly critical for prioritising oligomeric or fibrillar aSyn forms to maximise clinical impact. Advanced technologies, such as cryo-EM, mass spectrometry, and cutting-edge cellular models, will play a pivotal role in elucidating the structural basis of antibody–antigen interactions and guiding the optimisation of antibody engineering. Overcoming these challenges through innovative design and rigorous validation will be critical to unlocking the full therapeutic potential of antibody-based strategies. Such advancements promise transformative treatments that can mitigate disease progression and significantly improve patient outcomes.

Another approach includes developing anti-aggregation therapies (Figure 4). In this regard, a deeper understanding of aSyn structure and its cellular uptake is crucial for developing selective molecular inhibitors to mitigate aggregation [337] and prion-like propagation [338]. Recently, CBP/p300 inhibitors have been proposed as potential disruptors of pathological aSyn accumulation in dopaminergic neurons [339], while the BBB-penetrating inhibitor AZD3759 has demonstrated the ability to attenuate prion-like aSyn propagation in mice [340]. Moreover, small aromatic compounds such as SynuClean-D have been shown to inhibit aggregation across multiple aSyn strains [341,342]. Combining in silico and in vitro models has enabled the development of modern strategies for designing small molecules that target secondary nucleation, thereby further advancing therapeutic development [343]. Furthermore, recent studies suggest that promoting aSyn clearance through physiological reactivation of the perivascular fluid transport network, also known as the glymphatic system, may offer a novel therapeutic approach. The glymphatic system plays a crucial role in clearing neurotoxins, including aSyn, and its modulation could represent a viable strategy for reducing the aSyn burden in neurodegenerative diseases [344,345,346,347].

Nevertheless, another strategy involves the use of new technologies such as nanomedicine (the application of nanotechnology in medicine), which has achieved significant results in the development of selective enzyme-type catalytic nanostructures with properties for the removal of reactive oxygen species derived from the aggregation of aSyn, as well as with the capacity for the selective proteolysis of misfolded proteins [348]. Although some of these proposals are in the early stages, the deepening of our knowledge concerning the pathological processes of synucleinopathies and advances in nanomedicine could allow the development of potential treatments for these currently incurable diseases.

## 7. Discussion

The complex and multifaceted biology of aSyn has placed it at the centre of synucleinopathy research. However, significant challenges persist in fully elucidating its role in disease pathogenesis and progression. This review synthesizes insights into aSyn’s structure, aggregation dynamics, and cellular interactions, highlighting critical knowledge gaps and outlining future research and therapeutic directions. Addressing these challenges, the field can move closer to unravelling the complexities of aSyn biology and developing effective treatments for synucleinopathies.

A fundamental challenge in synucleinopathy research is deciphering the diverse aggregation states of aSyn and their effects on cellular function. aSyn aggregates, including oligomers, fibrils, and ribbons, adopt distinct conformations and exert varied biological effects, potentially functioning as “strains” contributing to disease heterogeneity [119,120,349,350]. Advanced methodologies, such as cryo-electron microscopy (cryo-EM) and single-molecule imaging, have significantly enhanced structural characterisation in both experimental models and human-derived samples. However, establishing clear links between specific structural forms, clinical phenotypes, and disease progression remains an urgent objective. Further refinement of these tools and their application in longitudinal studies could provide deeper insights into the mechanisms driving aSyn aggregation and its pathological diversity, facilitating the development of targeted therapeutics.

The second challenge centres on understanding the relationship between aSyn and cellular protein homeostasis mechanisms. Dysfunctions in chaperone-mediated autophagy, macroautophagy, and the ubiquitin–proteasome system contribute to aSyn accumulation and aggregation [87,89]. Recent studies have shown that activating the ubiquitin–proteasome system could dismantle disease-related proteins such as aSyn [351]. Emerging technologies, such as CRISPR-based gene-editing tools and high-throughput proteomics, offer promising avenues for identifying key molecular players involved in aSyn degradation [352,353,354]. Therapeutically targeting these pathways holds significant potential for restoring protein homeostasis and mitigating the neurodegenerative processes underlying synucleinopathies.

The third challenge lies in developing reliable biomarkers for early diagnosis and disease monitoring. The detection of aSyn in CSF, blood, and other biological fluids has shown considerable potential, particularly in identifying post-translationally modified species linked to pathology [320,324]. However, the lack of standardised and validated detection assays across laboratories remains a significant barrier to reproducibility and clinical translation. Advanced techniques, such as RT-QuiC and PMCA, have demonstrated exceptional sensitivity in detecting aggregated aSyn species [327,328]. Expanding these methodologies to longitudinal studies and at-risk populations could revolutionize early diagnosis and enhance disease monitoring, paving the way for more effective interventions.

The fourth challenge involves advancing in vivo imaging of aSyn pathology using PET tracers [355,356]. These tools present a transformative opportunity to visualise aSyn pathology in real time, offering critical insights into the spatial and temporal dynamics of disease progression. However, challenges such as BBB penetration and tracer specificity hinder their effectiveness [195,326]. Overcoming these limitations requires advances in molecular imaging techniques and the development of high-affinity, selective radioligands. Enhancing PET imaging capabilities will improve diagnostic accuracy and enable more precise monitoring of therapeutic responses in clinical settings.

The fifth challenge involves optimising immunotherapies targeting aSyn [357,358]. Conformation-specific antibodies have demonstrated promise in preclinical and clinical studies. However, their efficacy remains limited by challenges such as BBB penetration and specificity for pathological forms of aSyn [329,333,359]. Strategies to humanize antibodies, improve delivery mechanisms, and increase specificity for pathological forms are crucial. Complementary therapeutic approaches, including small-molecule inhibitors, peptides, and RNA-based interventions [360], can modulate aSyn aggregation and toxicity. Determining which aSyn species—oligomers, fibrils, or other forms—should be targeted remains a key question in optimising immunotherapeutic strategies.

The sixth challenge explores the complex interplay between peripheral immune responses, the gut microbiome, and central aSyn pathology. aSyn is a small, highly dynamic protein. Alterations in the gut microbiota have promoted aSyn aggregation and neuroinflammation, underscoring the gut–brain axis as a key modulator of disease progression [249,361,362]. Innovative therapeutic strategies, such as probiotics, prebiotics, faecal microbiota transplantation, dietary interventions, and gut-specific pharmacological agents, are being explored for their potential to influence these processes. Further research into the systemic interactions between peripheral and central mechanisms may yield novel insights into the pathogenesis of synucleinopathy, opening new avenues for targeted and holistic interventions.

The seventh challenge highlights the need for enhanced experimental and clinical models to study aSyn pathology. While transgenic animal models and patient-derived iPS cells have provided critical insights into aSyn aggregation and toxicity, they often fail to replicate key aspects of human pathology [273,282,363]. Novel approaches, such as CRISPR/Cas9-generated knock-in models and advanced 3D culture systems, could better capture disease-relevant processes and provide robust platforms for testing therapeutic interventions. Developing more physiologically relevant models will enhance the translational potential of preclinical research.

Another emerging area of interest involves cross-seeding mechanisms between distinct amyloidogenic proteins. Pre-existing tauopathies or amyloid-β deposits may catalyze α-synuclein aggregation through heterotypic seeding, potentially accelerating disease onset or modifying clinical trajectories. This inter-protein interaction underscores the complexity and overlap among neurodegenerative pathologies.

Addressing these challenges demands a multidisciplinary approach that combines cutting-edge technologies, robust experimental models, and clinical insights. Key priorities include unravelling the molecular triggers of aSyn aggregation, standardising and validating biomarker detection assays, advancing therapeutic innovation through immunotherapies and gene-editing tools, and investigating the systemic interactions that drive synucleinopathies. Collaborative efforts across scientific disciplines and the integration of emerging technologies will be vital to overcoming these barriers.

By framing aSyn research within these critical challenges, the field is poised to make substantial progress in understanding and treating synucleinopathies. A comprehensive and strategic approach will drive progress in developing effective treatments, offering renewed hope for improved patient outcomes in these debilitating disorders.

## Abbreviations

The following abbreviations are used in this manuscript:

BBB	Blood–Brain Barrier
CMA	Chaperone-Mediated Autophagy
CNS	Central Nervous System
CRISPR	Clustered Regularly Interspaced Short Palindromic Repeats
CSF	Cerebrospinal Fluid
DLB	Dementia with Lewy Bodies
ER	Endoplasmic Reticulum
GBA1	Glucocerebrosidase Beta 1
GCIs	Glial Cytoplasmic Inclusions
GWAS	Genome-Wide Association Study
IDP	Intrinsically Disordered Protein
LB	Lewy Body
LRRK2	Leucine-Rich Repeat Kinase 2
MAMs	Mitochondria-Associated Membranes
MRI	Magnetic Resonance Imaging
MSA	Multiple System Atrophy
NAC	Non-Amyloid Component
NMR	Nuclear Magnetic Resonance
PD	Parkinson’s Disease
PET	Positron Emission Tomography
PMCA	Protein Misfolding Cyclic Amplification
PrPC	Cellular Prion Protein
RT-QuiC	Real-Time Quaking-Induced Conversion
SAA	Seed Amplification Assay
SN	Substantia Nigra
UPS	Ubiquitin–Proteasome System

## References

[1] Fanciulli A., Wenning G.K. (2015). Multiple-system atrophy. N. Engl. J. Med..

[2] Poewe W., Stankovic I., Halliday G., Meissner W.G., Wenning G.K., Pellecchia M.T., Seppi K., Palma J.A., Kaufmann H. (2022). Multiple system atrophy. Nat. Rev. Dis. Primers.

[3] Gibbons C.H., Levine T., Adler C., Bellaire B., Wang N., Stohl J., Agarwal P., Aldridge G.M., Barboi A., Evidente V.G.H. (2024). Skin Biopsy Detection of Phosphorylated Alpha-Synuclein in Patients With Synucleinopathies. JAMA.

[4] Kalia L.V., Berg D., Kordower J.H., Shannon K.M., Taylor J.P., Cardoso F., Goldman J.G., Jeon B., Meissner W.G., Tijssen M.A.J. (2024). International Parkinson and Movement Disorder Society Viewpoint on Biological Frameworks of Parkinson’s Disease: Current Status and Future Directions. Mov. Disord..

[5] Maroteaux L., Campanelli J.T., Scheller R.H. (1988). Synuclein: A neuron-specific protein localized to the nucleus and presynaptic nerve terminal. J. Neurosci..

[6] Poulopoulos M., Levy O.A., Alcalay R.N. (2012). The neuropathology of genetic Parkinson’s disease. Mov. Disord..

[7] Spillantini M.G., Schmidt M.L., Lee V.M., Trojanowski J.Q., Jakes R., Goedert M. (1997). Alpha-synuclein in Lewy bodies. Nature.

[8] Spillantini M.G., Crowther R.A., Jakes R., Hasegawa M., Goedert M. (1998). Alpha-Synuclein in filamentous inclusions of Lewy bodies from Parkinson’s disease and dementia with lewy bodies. Proc. Natl. Acad. Sci. USA.

[9] Wakabayashi K., Yoshimoto M., Tsuji S., Takahashi H. (1998). Alpha-synuclein immunoreactivity in glial cytoplasmic inclusions in multiple system atrophy. Neurosci. Lett..

[10] Lau A., So R.W.L., Lau H.H.C., Sang J.C., Ruiz-Riquelme A., Fleck S.C., Stuart E., Menon S., Visanji N.P., Meisl G. (2020). Alpha-Synuclein strains target distinct brain regions and cell types. Nat. Neurosci..

[11] Shahnawaz M., Mukherjee A., Pritzkow S., Mendez N., Rabadia P., Liu X., Hu B., Schmeichel A., Singer W., Wu G. (2020). Discriminating alpha-synuclein strains in Parkinson’s disease and multiple system atrophy. Nature.

[12] Prusiner S.B., Woerman A.L., Mordes D.A., Watts J.C., Rampersaud R., Berry D.B., Patel S., Oehler A., Lowe J.K., Kravitz S.N. (2015). Evidence for alpha-synuclein prions causing multiple system atrophy in humans with parkinsonism. Proc. Natl. Acad. Sci. USA.

[13] Espay A.J., Vizcarra J.A., Marsili L., Lang A.E., Simon D.K., Merola A., Josephs K.A., Fasano A., Morgante F., Savica R. (2019). Revisiting protein aggregation as pathogenic in sporadic Parkinson and Alzheimer diseases. Neurology.

[14] Ahn B.H., Rhim H., Kim S.Y., Sung Y.M., Lee M.Y., Choi J.Y., Wolozin B., Chang J.S., Lee Y.H., Kwon T.K. (2002). Alpha-Synuclein interacts with phospholipase D isozymes and inhibits pervanadate-induced phospholipase D activation in human embryonic kidney-293 cells. J. Biol. Chem..

[15] Emamzadeh F.N. (2016). Alpha-synuclein structure, functions, and interactions. J. Res. Med. Sci..

[16] Giasson B.I., Murray I.V., Trojanowski J.Q., Lee V.M. (2001). A hydrophobic stretch of 12 amino acid residues in the middle of alpha-synuclein is essential for filament assembly. J. Biol. Chem..

[17] Eliezer D., Kutluay E., Bussell R., Browne G. (2001). Conformational properties of alpha-synuclein in its free and lipid-associated states. J. Mol. Biol..

[18] Bodner C.R., Dobson C.M., Bax A. (2009). Multiple tight phospholipid-binding modes of alpha-synuclein revealed by solution NMR spectroscopy. J. Mol. Biol..

[19] Uversky V.N. (2003). A protein-chameleon: Conformational plasticity of alpha-synuclein, a disordered protein involved in neurodegenerative disorders. J. Biomol. Struct. Dyn..

[20] Meade R.M., Fairlie D.P., Mason J.M. (2019). Alpha-synuclein structure and Parkinson’s disease—Lessons and emerging principles. Mol. Neurodegener..

[21] Mantsyzov A.B., Maltsev A.S., Ying J., Shen Y., Hummer G., Bax A. (2014). A maximum entropy approach to the study of residue-specific backbone angle distributions in alpha-synuclein, an intrinsically disordered protein. Protein Sci..

[22] Theillet F.X., Binolfi A., Bekei B., Martorana A., Rose H.M., Stuiver M., Verzini S., Lorenz D., van Rossum M., Goldfarb D. (2016). Structural disorder of monomeric alpha-synuclein persists in mammalian cells. Nature.

[23] Maltsev A.S., Ying J., Bax A. (2012). Impact of N-terminal acetylation of alpha-synuclein on its random coil and lipid binding properties. Biochemistry.

[24] Kjaergaard M., Brander S., Poulsen F.M. (2011). Random coil chemical shift for intrinsically disordered proteins: Effects of temperature and pH. J. Biomol. NMR.

[25] Galvagnion C., Brown J.W., Ouberai M.M., Flagmeier P., Vendruscolo M., Buell A.K., Sparr E., Dobson C.M. (2016). Chemical properties of lipids strongly affect the kinetics of the membrane-induced aggregation of alpha-synuclein. Proc. Natl. Acad. Sci. USA.

[26] Galvagnion C. (2017). The Role of Lipids Interacting with alpha-Synuclein in the Pathogenesis of Parkinson’s Disease. J. Park. Dis..

[27] Musteikyte G., Jayaram A.K., Xu C.K., Vendruscolo M., Krainer G., Knowles T.P.J. (2021). Interactions of alpha-synuclein oligomers with lipid membranes. Biochim. Biophys. Acta Biomembr..

[28] Kurouski D. (2023). Elucidating the Role of Lipids in the Aggregation of Amyloidogenic Proteins. Acc. Chem. Res..

[29] Heo P., Pincet F. (2020). Freezing and piercing of in vitro asymmetric plasma membrane by alpha-synuclein. Commun. Biol..

[30] Iyer A., Claessens M. (2019). Disruptive membrane interactions of alpha-synuclein aggregates. Biochim. Biophys. Acta Proteins Proteom..

[31] Ramirez J., Pancoe S.X., Rhoades E., Petersson E.J. (2023). The Effects of Lipids on alpha-Synuclein Aggregation In Vitro. Biomolecules.

[32] de Boni L., Wallis A., Hays Watson A., Ruiz-Riquelme A., Leyland L.A., Bourinaris T., Hannaway N., Wullner U., Peters O., Priller J. (2024). Aggregation-resistant alpha-synuclein tetramers are reduced in the blood of Parkinson’s patients. EMBO Mol. Med..

[33] Logan T., Bendor J., Toupin C., Thorn K., Edwards R.H. (2017). Alpha-Synuclein promotes dilation of the exocytotic fusion pore. Nat. Neurosci..

[34] Calabresi P., Di Lazzaro G., Marino G., Campanelli F., Ghiglieri V. (2023). Advances in understanding the function of alpha-synuclein: Implications for Parkinson’s disease. Brain.

[35] Sharma M., Burre J. (2023). Alpha-Synuclein in synaptic function and dysfunction. Trends Neurosci..

[36] Parra-Rivas L.A., Madhivanan K., Aulston B.D., Wang L., Prakashchand D.D., Boyer N.P., Saia-Cereda V.M., Branes-Guerrero K., Pizzo D.P., Bagchi P. (2023). Serine-129 phosphorylation of alpha-synuclein is an activity-dependent trigger for physiologic protein-protein interactions and synaptic function. Neuron.

[37] Ramalingam N., Jin S.X., Moors T.E., Fonseca-Ornelas L., Shimanaka K., Lei S., Cam H.P., Watson A.H., Brontesi L., Ding L. (2023). Dynamic physiological alpha-synuclein S129 phosphorylation is driven by neuronal activity. NPJ Park. Dis..

[38] Bartels T., Choi J.G., Selkoe D.J. (2011). Alpha-Synuclein occurs physiologically as a helically folded tetramer that resists aggregation. Nature.

[39] Park H., Kam T.I., Dawson V.L., Dawson T.M. (2025). Alpha-Synuclein pathology as a target in neurodegenerative diseases. Nat. Rev. Neurol..

[40] Bernal-Conde L.D., Ramos-Acevedo R., Reyes-Hernandez M.A., Balbuena-Olvera A.J., Morales-Moreno I.D., Arguero-Sanchez R., Schule B., Guerra-Crespo M. (2019). Alpha-Synuclein Physiology and Pathology: A Perspective on Cellular Structures and Organelles. Front. Neurosci..

[41] Fortin D.L., Troyer M.D., Nakamura K., Kubo S., Anthony M.D., Edwards R.H. (2004). Lipid rafts mediate the synaptic localization of alpha-synuclein. J. Neurosci..

[42] Man W.K., Tahirbegi B., Vrettas M.D., Preet S., Ying L., Vendruscolo M., De Simone A., Fusco G. (2021). The docking of synaptic vesicles on the presynaptic membrane induced by alpha-synuclein is modulated by lipid composition. Nat. Commun..

[43] Chandra S., Chen X., Rizo J., Jahn R., Sudhof T.C. (2003). A broken alpha -helix in folded alpha -Synuclein. J. Biol. Chem..

[44] Burre J., Sharma M., Tsetsenis T., Buchman V., Etherton M.R., Sudhof T.C. (2010). Alpha-synuclein promotes SNARE-complex assembly in vivo and in vitro. Science.

[45] Ghio S., Kamp F., Cauchi R., Giese A., Vassallo N. (2016). Interaction of alpha-synuclein with biomembranes in Parkinson’s disease--role of cardiolipin. Prog. Lipid Res..

[46] Di Maio R., Barrett P.J., Hoffman E.K., Barrett C.W., Zharikov A., Borah A., Hu X., McCoy J., Chu C.T., Burton E.A. (2016). Alpha-Synuclein binds to TOM20 and inhibits mitochondrial protein import in Parkinson’s disease. Sci. Transl. Med..

[47] Colla E., Coune P., Liu Y., Pletnikova O., Troncoso J.C., Iwatsubo T., Schneider B.L., Lee M.K. (2012). Endoplasmic reticulum stress is important for the manifestations of alpha-synucleinopathy in vivo. J. Neurosci..

[48] Mazzulli J.R., Zunke F., Isacson O., Studer L., Krainc D. (2016). Alpha-Synuclein-induced lysosomal dysfunction occurs through disruptions in protein trafficking in human midbrain synucleinopathy models. Proc. Natl. Acad. Sci. USA.

[49] Grassi D., Howard S., Zhou M., Diaz-Perez N., Urban N.T., Guerrero-Given D., Kamasawa N., Volpicelli-Daley L.A., LoGrasso P., Lasmezas C.I. (2018). Identification of a highly neurotoxic alpha-synuclein species inducing mitochondrial damage and mitophagy in Parkinson’s disease. Proc. Natl. Acad. Sci. USA.

[50] Goncalves S., Outeiro T.F. (2013). Assessing the subcellular dynamics of alpha-synuclein using photoactivation microscopy. Mol. Neurobiol..

[51] Schaser A.J., Osterberg V.R., Dent S.E., Stackhouse T.L., Wakeham C.M., Boutros S.W., Weston L.J., Owen N., Weissman T.A., Luna E. (2019). Alpha-synuclein is a DNA binding protein that modulates DNA repair with implications for Lewy body disorders. Sci. Rep..

[52] Barbour R., Kling K., Anderson J.P., Banducci K., Cole T., Diep L., Fox M., Goldstein J.M., Soriano F., Seubert P. (2008). Red blood cells are the major source of alpha-synuclein in blood. Neurodegener. Dis..

[53] Pan-Montojo F., Anichtchik O., Dening Y., Knels L., Pursche S., Jung R., Jackson S., Gille G., Spillantini M.G., Reichmann H. (2010). Progression of Parkinson’s disease pathology is reproduced by intragastric administration of rotenone in mice. PLoS ONE.

[54] Pan-Montojo F., Schwarz M., Winkler C., Arnhold M., O’Sullivan G.A., Pal A., Said J., Marsico G., Verbavatz J.M., Rodrigo-Angulo M. (2012). Environmental toxins trigger PD-like progression via increased alpha-synuclein release from enteric neurons in mice. Sci. Rep..

[55] Engelender S., Kaminsky Z., Guo X., Sharp A.H., Amaravi R.K., Kleiderlein J.J., Margolis R.L., Troncoso J.C., Lanahan A.A., Worley P.F. (1999). Synphilin-1 associates with alpha-synuclein and promotes the formation of cytosolic inclusions. Nat. Genet..

[56] Guerreiro P.S., Huang Y., Gysbers A., Cheng D., Gai W.P., Outeiro T.F., Halliday G.M. (2013). LRRK2 interactions with alpha-synuclein in Parkinson’s disease brains and in cell models. J. Mol. Med..

[57] Qing H., Zhang Y., Deng Y., McGeer E.G., McGeer P.L. (2009). Lrrk2 interaction with alpha-synuclein in diffuse Lewy body disease. Biochem. Biophys. Res. Commun..

[58] Ejlerskov P., Rasmussen I., Nielsen T.T., Bergstrom A.L., Tohyama Y., Jensen P.H., Vilhardt F. (2013). Tubulin polymerization-promoting protein (TPPP/p25alpha) promotes unconventional secretion of alpha-synuclein through exophagy by impairing autophagosome-lysosome fusion. J. Biol. Chem..

[59] Zondler L., Miller-Fleming L., Repici M., Goncalves S., Tenreiro S., Rosado-Ramos R., Betzer C., Straatman K.R., Jensen P.H., Giorgini F. (2014). DJ-1 interactions with alpha-synuclein attenuate aggregation and cellular toxicity in models of Parkinson’s disease. Cell Death Dis..

[60] Gitler A.D., Chesi A., Geddie M.L., Strathearn K.E., Hamamichi S., Hill K.J., Caldwell K.A., Caldwell G.A., Cooper A.A., Rochet J.C. (2009). Alpha-synuclein is part of a diverse and highly conserved interaction network that includes PARK9 and manganese toxicity. Nat. Genet..

[61] Lopes da Fonseca T., Pinho R., Outeiro T.F. (2016). A familial ATP13A2 mutation enhances alpha-synuclein aggregation and promotes cell death. Hum. Mol. Genet..

[62] Nakhjavani M., Morteza A., Khajeali L., Esteghamati A., Khalilzadeh O., Asgarani F., Outeiro T.F. (2010). Increased serum HSP70 levels are associated with the duration of diabetes. Cell Stress Chaperones.

[63] Outeiro T.F., Klucken J., Strathearn K.E., Liu F., Nguyen P., Rochet J.C., Hyman B.T., McLean P.J. (2006). Small heat shock proteins protect against alpha-synuclein-induced toxicity and aggregation. Biochem. Biophys. Res. Commun..

[64] Breda C., Nugent M.L., Estranero J.G., Kyriacou C.P., Outeiro T.F., Steinert J.R., Giorgini F. (2015). Rab11 modulates alpha-synuclein-mediated defects in synaptic transmission and behaviour. Hum. Mol. Genet..

[65] Chutna O., Goncalves S., Villar-Pique A., Guerreiro P., Marijanovic Z., Mendes T., Ramalho J., Emmanouilidou E., Ventura S., Klucken J. (2014). The small GTPase Rab11 co-localizes with alpha-synuclein in intracellular inclusions and modulates its aggregation, secretion and toxicity. Hum. Mol. Genet..

[66] Ihse E., Yamakado H., van Wijk X.M., Lawrence R., Esko J.D., Masliah E. (2017). Cellular internalization of alpha-synuclein aggregates by cell surface heparan sulfate depends on aggregate conformation and cell type. Sci. Rep..

[67] Pinho R., Paiva I., Jercic K.G., Fonseca-Ornelas L., Gerhardt E., Fahlbusch C., Garcia-Esparcia P., Kerimoglu C., Pavlou M.A.S., Villar-Pique A. (2019). Nuclear localization and phosphorylation modulate pathological effects of alpha-synuclein. Hum. Mol. Genet..

[68] Jo E., McLaurin J., Yip C.M., St George-Hyslop P., Fraser P.E. (2000). Alpha-Synuclein membrane interactions and lipid specificity. J. Biol. Chem..

[69] Chen R.H.C., Wislet-Gendebien S., Samuel F., Visanji N.P., Zhang G., Marsilio D., Langman T., Fraser P.E., Tandon A. (2013). Alpha-Synuclein membrane association is regulated by the Rab3a recycling machinery and presynaptic activity. J. Biol. Chem..

[70] Zaltieri M., Grigoletto J., Longhena F., Navarria L., Favero G., Castrezzati S., Colivicchi M.A., Della Corte L., Rezzani R., Pizzi M. (2015). Alpha-synuclein and synapsin III cooperatively regulate synaptic function in dopamine neurons. J. Cell Sci..

[71] Butler B., Saha K., Rana T., Becker J.P., Sambo D., Davari P., Goodwin J.S., Khoshbouei H. (2015). Dopamine Transporter Activity Is Modulated by alpha-Synuclein. J. Biol. Chem..

[72] Baptista M.J., O’Farrell C., Daya S., Ahmad R., Miller D.W., Hardy J., Farrer M.J., Cookson M.R. (2003). Co-ordinate transcriptional regulation of dopamine synthesis genes by alpha-synuclein in human neuroblastoma cell lines. J. Neurochem..

[73] Martinez J.H., Fuentes F., Vanasco V., Alvarez S., Alaimo A., Cassina A., Coluccio Leskow F., Velazquez F. (2018). Alpha-synuclein mitochondrial interaction leads to irreversible translocation and complex I impairment. Arch. Biochem. Biophys..

[74] Devi L., Raghavendran V., Prabhu B.M., Avadhani N.G., Anandatheerthavarada H.K. (2008). Mitochondrial import and accumulation of alpha-synuclein impair complex I in human dopaminergic neuronal cultures and Parkinson disease brain. J. Biol. Chem..

[75] Choi M.L., Chappard A., Singh B.P., Maclachlan C., Rodrigues M., Fedotova E.I., Berezhnov A.V., De S., Peddie C.J., Athauda D. (2022). Pathological structural conversion of alpha-synuclein at the mitochondria induces neuronal toxicity. Nat. Neurosci..

[76] Negi S., Khurana N., Duggal N. (2024). The misfolding mystery: Alpha-synuclein and the pathogenesis of Parkinson’s disease. Neurochem. Int..

[77] Dou L., Xu Z., Xu J., Su C., Pieper A.A., Zhu X., Leverenz J.B., Wang F., Cummings J., Cheng F. (2025). A network-based systems genetics framework identifies pathobiology and drug repurposing in Parkinson’s disease. NPJ Park. Dis..

[78] van Diggelen F., Frank S.A., Somavarapu A.K., Scavenius C., Apetri M.M., Nielsen J., Tepper A., Enghild J.J., Otzen D.E. (2020). The interactome of stabilized alpha-synuclein oligomers and neuronal proteins. FEBS J..

[79] Estaun-Panzano J., Arotcarena M.L., Bezard E. (2023). Monitoring alpha-synuclein aggregation. Neurobiol. Dis..

[80] Sulzer D., Edwards R.H. (2019). The physiological role of alpha-synuclein and its relationship to Parkinson’s Disease. J. Neurochem..

[81] Balupuri A., Choi K.E., Kang N.S. (2019). Computational insights into the role of alpha-strand/sheet in aggregation of alpha-synuclein. Sci. Rep..

[82] Mahur P., Sharma A., Jahan G., Adithya S.G., Kumar Singh A., Muthukumaran J., Jain M. (2024). Understanding Genetic Risks: Computational Exploration of Human beta-Synuclein nsSNPs and their Potential Impact on Structural Alteration. Neurosci. Lett..

[83] Sang J.C., Hidari E., Meisl G., Ranasinghe R.T., Spillantini M.G., Klenerman D. (2021). Super-resolution imaging reveals alpha-synuclein seeded aggregation in SH-SY5Y cells. Commun. Biol..

[84] Kuang Y., Mao H., Huang X., Chen M., Dai W., Gan T., Wang J., Sun H., Lin H., Liu Q. (2024). Alpha-Synuclein seeding amplification assays for diagnosing synucleinopathies: An innovative tool in clinical implementation. Transl. Neurodegener..

[85] Vogiatzi T., Xilouri M., Vekrellis K., Stefanis L. (2008). Wild type alpha-synuclein is degraded by chaperone-mediated autophagy and macroautophagy in neuronal cells. J. Biol. Chem..

[86] Sala G., Marinig D., Arosio A., Ferrarese C. (2016). Role of Chaperone-Mediated Autophagy Dysfunctions in the Pathogenesis of Parkinson’s Disease. Front. Mol. Neurosci..

[87] Martinez-Vicente M., Talloczy Z., Kaushik S., Massey A.C., Mazzulli J., Mosharov E.V., Hodara R., Fredenburg R., Wu D.C., Follenzi A. (2008). Dopamine-modified alpha-synuclein blocks chaperone-mediated autophagy. J. Clin. Investig..

[88] Salvador N., Aguado C., Horst M., Knecht E. (2000). Import of a cytosolic protein into lysosomes by chaperone-mediated autophagy depends on its folding state. J. Biol. Chem..

[89] Liu M., Li X.L., Hassel B.A. (2003). Proteasomes modulate conjugation to the ubiquitin-like protein, ISG15. J. Biol. Chem..

[90] Emmanouilidou E., Melachroinou K., Roumeliotis T., Garbis S.D., Ntzouni M., Margaritis L.H., Stefanis L., Vekrellis K. (2010). Cell-produced alpha-synuclein is secreted in a calcium-dependent manner by exosomes and impacts neuronal survival. J. Neurosci..

[91] Dahmene M., Berard M., Oueslati A. (2017). Dissecting the Molecular Pathway Involved in PLK2 Kinase-mediated alpha-Synuclein-selective Autophagic Degradation. J. Biol. Chem..

[92] Fares M.B., Ait-Bouziad N., Dikiy I., Mbefo M.K., Jovicic A., Kiely A., Holton J.L., Lee S.J., Gitler A.D., Eliezer D. (2014). The novel Parkinson’s disease linked mutation G51D attenuates in vitro aggregation and membrane binding of alpha-synuclein, and enhances its secretion and nuclear localization in cells. Hum. Mol. Genet..

[93] Khalaf O., Fauvet B., Oueslati A., Dikiy I., Mahul-Mellier A.L., Ruggeri F.S., Mbefo M.K., Vercruysse F., Dietler G., Lee S.J. (2014). The H50Q mutation enhances alpha-synuclein aggregation, secretion, and toxicity. J. Biol. Chem..

[94] Loria F., Vargas J.Y., Bousset L., Syan S., Salles A., Melki R., Zurzolo C. (2017). Alpha-Synuclein transfer between neurons and astrocytes indicates that astrocytes play a role in degradation rather than in spreading. Acta Neuropathol..

[95] Karpowicz R.J., Haney C.M., Mihaila T.S., Sandler R.M., Petersson E.J., Lee V.M. (2017). Selective imaging of internalized proteopathic alpha-synuclein seeds in primary neurons reveals mechanistic insight into transmission of synucleinopathies. J. Biol. Chem..

[96] Lee H.J., Suk J.E., Bae E.J., Lee S.J. (2008). Clearance and deposition of extracellular alpha-synuclein aggregates in microglia. Biochem. Biophys. Res. Commun..

[97] Nathan J.A., Kim H.T., Ting L., Gygi S.P., Goldberg A.L. (2013). Why do cellular proteins linked to K63-polyubiquitin chains not associate with proteasomes?. EMBO J..

[98] Rott R., Szargel R., Haskin J., Shani V., Shainskaya A., Manov I., Liani E., Avraham E., Engelender S. (2008). Monoubiquitylation of alpha-synuclein by seven in absentia homolog (SIAH) promotes its aggregation in dopaminergic cells. J. Biol. Chem..

[99] Vicente Miranda H., Cassio R., Correia-Guedes L., Gomes M.A., Chegao A., Miranda E., Soares T., Coelho M., Rosa M.M., Ferreira J.J. (2017). Posttranslational modifications of blood-derived alpha-synuclein as biochemical markers for Parkinson’s disease. Sci. Rep..

[100] Oueslati A. (2016). Implication of Alpha-Synuclein Phosphorylation at S129 in Synucleinopathies: What Have We Learned in the Last Decade?. J. Park. Dis..

[101] Cullen V., Lindfors M., Ng J., Paetau A., Swinton E., Kolodziej P., Boston H., Saftig P., Woulfe J., Feany M.B. (2009). Cathepsin D expression level affects alpha-synuclein processing, aggregation, and toxicity in vivo. Mol. Brain.

[102] Ebrahimi-Fakhari D., Cantuti-Castelvetri I., Fan Z., Rockenstein E., Masliah E., Hyman B.T., McLean P.J., Unni V.K. (2011). Distinct roles in vivo for the ubiquitin-proteasome system and the autophagy-lysosomal pathway in the degradation of alpha-synuclein. J. Neurosci..

[103] Friedman L.G., Lachenmayer M.L., Wang J., He L., Poulose S.M., Komatsu M., Holstein G.R., Yue Z. (2012). Disrupted autophagy leads to dopaminergic axon and dendrite degeneration and promotes presynaptic accumulation of alpha-synuclein and LRRK2 in the brain. J. Neurosci..

[104] Gathings A., Zaman V., Banik N.L., Haque A. (2024). Insights into Calpain Activation and Rho-ROCK Signaling in Parkinson’s Disease and Aging. Biomedicines.

[105] Absalyamova M., Traktirov D., Burdinskaya V., Artemova V., Muruzheva Z., Karpenko M. (2025). Molecular basis of the development of Parkinson’s disease. Neuroscience.

[106] Behl T., Kaur G., Sehgal A., Bhardwaj S., Singh S., Buhas C., Judea-Pusta C., Uivarosan D., Munteanu M.A., Bungau S. (2021). Multifaceted Role of Matrix Metalloproteinases in Neurodegenerative Diseases: Pathophysiological and Therapeutic Perspectives. Int. J. Mol. Sci..

[107] Xilouri M., Brekk O.R., Stefanis L. (2013). Alpha-Synuclein and protein degradation systems: A reciprocal relationship. Mol. Neurobiol..

[108] Lopes da Fonseca T., Villar-Pique A., Outeiro T.F. (2015). The Interplay between Alpha-Synuclein Clearance and Spreading. Biomolecules.

[109] Sorrentino Z.A., Giasson B.I., Chakrabarty P. (2019). Alpha-Synuclein and astrocytes: Tracing the pathways from homeostasis to neurodegeneration in Lewy body disease. Acta Neuropathol..

[110] Mavroeidi P., Xilouri M. (2021). Neurons and Glia Interplay in alpha-Synucleinopathies. Int. J. Mol. Sci..

[111] Oliveira L.M.A., Gasser T., Edwards R., Zweckstetter M., Melki R., Stefanis L., Lashuel H.A., Sulzer D., Vekrellis K., Halliday G.M. (2021). Alpha-synuclein research: Defining strategic moves in the battle against Parkinson’s disease. NPJ Park. Dis..

[112] Sahoo S., Padhy A.A., Kumari V., Mishra P. (2022). Role of Ubiquitin-Proteasome and Autophagy-Lysosome Pathways in Alpha-Synuclein Aggregate Clearance. Mol. Neurobiol..

[113] Masuda-Suzukake M., Nonaka T., Hosokawa M., Oikawa T., Arai T., Akiyama H., Mann D.M., Hasegawa M. (2013). Prion-like spreading of pathological alpha-synuclein in brain. Brain.

[114] Peng C., Gathagan R.J., Covell D.J., Medellin C., Stieber A., Robinson J.L., Zhang B., Pitkin R.M., Olufemi M.F., Luk K.C. (2018). Cellular milieu imparts distinct pathological alpha-synuclein strains in alpha-synucleinopathies. Nature.

[115] Gurry T., Ullman O., Fisher C.K., Perovic I., Pochapsky T., Stultz C.M. (2013). The dynamic structure of alpha-synuclein multimers. J. Am. Chem. Soc..

[116] Caughey B., Lansbury P.T. (2003). Protofibrils, pores, fibrils, and neurodegeneration: Separating the responsible protein aggregates from the innocent bystanders. Annu. Rev. Neurosci..

[117] Santos J., Pallares I., Ventura S. (2024). A glimpse into the structural properties of alpha-synuclein oligomers. Biofactors.

[118] Li X., Dong C., Hoffmann M., Garen C.R., Cortez L.M., Petersen N.O., Woodside M.T. (2019). Early stages of aggregation of engineered alpha-synuclein monomers and oligomers in solution. Sci. Rep..

[119] Bousset L., Pieri L., Ruiz-Arlandis G., Gath J., Jensen P.H., Habenstein B., Madiona K., Olieric V., Bockmann A., Meier B.H. (2013). Structural and functional characterization of two alpha-synuclein strains. Nat. Commun..

[120] Guo J.L., Covell D.J., Daniels J.P., Iba M., Stieber A., Zhang B., Riddle D.M., Kwong L.K., Xu Y., Trojanowski J.Q. (2013). Distinct alpha-synuclein strains differentially promote tau inclusions in neurons. Cell.

[121] Dening Y., Strassl T., Ruf V., Dirscherl P., Chovsepian A., Stievenard A., Khairnar A., Schmidt F., Giesert F., Herms J. (2022). Toxicity of extracellular alpha-synuclein is independent of intracellular alpha-synuclein. Sci. Rep..

[122] Kim C., Lv G., Lee J.S., Jung B.C., Masuda-Suzukake M., Hong C.S., Valera E., Lee H.J., Paik S.R., Hasegawa M. (2016). Exposure to bacterial endotoxin generates a distinct strain of alpha-synuclein fibril. Sci. Rep..

[123] Bernal-Conde L.D., Pena-Martinez V., Morato-Torres C.A., Ramos-Acevedo R., Arias-Carrion O., Padilla-Godinez F.J., Delgado-Gonzalez A., Palomero-Rivero M., Collazo-Navarrete O., Soto-Rojas L.O. (2024). Alpha-Synuclein Gene Alterations Modulate Tyrosine Hydroxylase in Human iPSC-Derived Neurons in a Parkinson’s Disease Animal Model. Life.

[124] Tanudjojo B., Shaikh S.S., Fenyi A., Bousset L., Agarwal D., Marsh J., Zois C., Heman-Ackah S., Fischer R., Sims D. (2021). Phenotypic manifestation of alpha-synuclein strains derived from Parkinson’s disease and multiple system atrophy in human dopaminergic neurons. Nat. Commun..

[125] Pancoe S.X., Wang Y.J., Shimogawa M., Perez R.M., Giannakoulias S., Petersson E.J. (2022). Effects of Mutations and Post-Translational Modifications on Alpha-Synuclein In Vitro Aggregation. J. Mol. Biol..

[126] Suthar S.K., Lee S.Y. (2023). Truncation or proteolysis of alpha-synuclein in Parkinsonism. Ageing Res. Rev..

[127] Schepers J., Loser T., Behl C. (2024). Lipids and Alpha-Synuclein: Adding further variables to the equation. Front. Mol. Biosci..

[128] Moors T.E., Maat C.A., Niedieker D., Mona D., Petersen D., Timmermans-Huisman E., Kole J., El-Mashtoly S.F., Spycher L., Zago W. (2021). The subcellular arrangement of alpha-synuclein proteoforms in the Parkinson’s disease brain as revealed by multicolor STED microscopy. Acta Neuropathol..

[129] Shahmoradian S.H., Lewis A.J., Genoud C., Hench J., Moors T.E., Navarro P.P., Castano-Diez D., Schweighauser G., Graff-Meyer A., Goldie K.N. (2019). Lewy pathology in Parkinson’s disease consists of crowded organelles and lipid membranes. Nat. Neurosci..

[130] Weihofen A., Liu Y., Arndt J.W., Huy C., Quan C., Smith B.A., Baeriswyl J.L., Cavegn N., Senn L., Su L. (2019). Development of an aggregate-selective, human-derived alpha-synuclein antibody BIIB054 that ameliorates disease phenotypes in Parkinson’s disease models. Neurobiol. Dis..

[131] Guerrero-Ferreira R., Taylor N.M., Mona D., Ringler P., Lauer M.E., Riek R., Britschgi M., Stahlberg H. (2018). Cryo-EM structure of alpha-synuclein fibrils. eLife.

[132] Tuttle M.D., Comellas G., Nieuwkoop A.J., Covell D.J., Berthold D.A., Kloepper K.D., Courtney J.M., Kim J.K., Barclay A.M., Kendall A. (2016). Solid-state NMR structure of a pathogenic fibril of full-length human alpha-synuclein. Nat. Struct. Mol. Biol..

[133] Anagnostou D., Sfakianaki G., Melachroinou K., Soutos M., Constantinides V., Vaikath N., Tsantzali I., Paraskevas G.P., Agnaf O.E., Vekrellis K. (2023). Assessment of Aggregated and Exosome-Associated alpha-Synuclein in Brain Tissue and Cerebrospinal Fluid Using Specific Immunoassays. Diagnostics.

[134] Kapsali I., Brinia M.E., Constantinides V.C. (2024). Cerebrospinal Fluid Total, Phosphorylated and Oligomeric A-Synuclein in Parkinson’s Disease: A Systematic Review, Meta-Analysis and Meta-Regression Study. Biomedicines.

[135] Zalon A.J., Quiriconi D.J., Pitcairn C., Mazzulli J.R. (2024). Alpha-Synuclein: Multiple pathogenic roles in trafficking and proteostasis pathways in Parkinson’s disease. Neuroscientist.

[136] Vadukul D.M., Papp M., Thrush R.J., Wang J., Jin Y., Arosio P., Aprile F.A. (2023). Alpha-Synuclein Aggregation Is Triggered by Oligomeric Amyloid-beta 42 via Heterogeneous Primary Nucleation. J. Am. Chem. Soc..

[137] Chau E., Kim J.R. (2022). Alpha-synuclein-assisted oligomerization of beta-amyloid (1–42). Arch. Biochem. Biophys..

[138] Singh B., Covelo A., Martell-Martinez H., Nanclares C., Sherman M.A., Okematti E., Meints J., Teravskis P.J., Gallardo C., Savonenko A.V. (2019). Tau is required for progressive synaptic and memory deficits in a transgenic mouse model of alpha-synucleinopathy. Acta Neuropathol..

[139] Riedel M., Goldbaum O., Richter-Landsberg C. (2009). Alpha-Synuclein promotes the recruitment of tau to protein inclusions in oligodendroglial cells: Effects of oxidative and proteolytic stress. J. Mol. Neurosci..

[140] Pan L., Li C., Meng L., Tian Y., He M., Yuan X., Zhang G., Zhang Z., Xiong J., Chen G. (2022). Tau accelerates alpha-synuclein aggregation and spreading in Parkinson’s disease. Brain.

[141] Magalhaes P., Lashuel H.A. (2022). Opportunities and challenges of alpha-synuclein as a potential biomarker for Parkinson’s disease and other synucleinopathies. NPJ Park. Dis..

[142] Ferreira D.G., Temido-Ferreira M., Vicente Miranda H., Batalha V.L., Coelho J.E., Szego E.M., Marques-Morgado I., Vaz S.H., Rhee J.S., Schmitz M. (2017). alpha-synuclein interacts with PrP(C) to induce cognitive impairment through mGluR5 and NMDAR2B. Nat. Neurosci..

[143] Mao X., Ou M.T., Karuppagounder S.S., Kam T.I., Yin X., Xiong Y., Ge P., Umanah G.E., Brahmachari S., Shin J.H. (2016). Pathological alpha-synuclein transmission initiated by binding lymphocyte-activation gene 3. Science.

[144] Gonzalez N., Arcos-Lopez T., Konig A., Quintanar L., Menacho Marquez M., Outeiro T.F., Fernandez C.O. (2019). Effects of alpha-synuclein post-translational modifications on metal binding. J. Neurochem..

[145] Moons R., Konijnenberg A., Mensch C., Van Elzen R., Johannessen C., Maudsley S., Lambeir A.M., Sobott F. (2020). Metal ions shape alpha-synuclein. Sci. Rep..

[146] Li Y., Yang C., Wang S., Yang D., Zhang Y., Xu L., Ma L., Zheng J., Petersen R.B., Zheng L. (2020). Copper and iron ions accelerate the prion-like propagation of alpha-synuclein: A vicious cycle in Parkinson’s disease. Int. J. Biol. Macromol..

[147] Bisaglia M., Tessari I., Mammi S., Bubacco L. (2009). Interaction between alpha-synuclein and metal ions, still looking for a role in the pathogenesis of Parkinson’s disease. NeuroMol. Med..

[148] Binolfi A., Rasia R.M., Bertoncini C.W., Ceolin M., Zweckstetter M., Griesinger C., Jovin T.M., Fernandez C.O. (2006). Interaction of alpha-synuclein with divalent metal ions reveals key differences: A link between structure, binding specificity and fibrillation enhancement. J. Am. Chem. Soc..

[149] Rasia R.M., Bertoncini C.W., Marsh D., Hoyer W., Cherny D., Zweckstetter M., Griesinger C., Jovin T.M., Fernandez C.O. (2005). Structural characterization of copper(II) binding to alpha-synuclein: Insights into the bioinorganic chemistry of Parkinson’s disease. Proc. Natl. Acad. Sci. USA.

[150] Lowe R., Pountney D.L., Jensen P.H., Gai W.P., Voelcker N.H. (2004). Calcium(II) selectively induces alpha-synuclein annular oligomers via interaction with the C-terminal domain. Protein Sci..

[151] Bras I.C., Dominguez-Meijide A., Gerhardt E., Koss D., Lazaro D.F., Santos P.I., Vasili E., Xylaki M., Outeiro T.F. (2020). Synucleinopathies: Where we are and where we need to go. J. Neurochem..

[152] Ghanem S.S., Majbour N.K., Vaikath N.N., Ardah M.T., Erskine D., Jensen N.M., Fayyad M., Sudhakaran I.P., Vasili E., Melachroinou K. (2022). Alpha-Synuclein phosphorylation at serine 129 occurs after initial protein deposition and inhibits seeded fibril formation and toxicity. Proc. Natl. Acad. Sci. USA.

[153] Paleologou K.E., Oueslati A., Shakked G., Rospigliosi C.C., Kim H.Y., Lamberto G.R., Fernandez C.O., Schmid A., Chegini F., Gai W.P. (2010). Phosphorylation at S87 is enhanced in synucleinopathies, inhibits alpha-synuclein oligomerization, and influences synuclein-membrane interactions. J. Neurosci..

[154] Xiong Y., Yu J. (2018). Modeling Parkinson’s Disease in Drosophila: What Have We Learned for Dominant Traits?. Front. Neurol..

[155] Abeyawardhane D.L., Fernandez R.D., Heitger D.R., Crozier M.K., Wolver J.C., Lucas H.R. (2018). Copper Induced Radical Dimerization of alpha-Synuclein Requires Histidine. J. Am. Chem. Soc..

[156] de Oliveira R.M., Vicente Miranda H., Francelle L., Pinho R., Szego E.M., Martinho R., Munari F., Lazaro D.F., Moniot S., Guerreiro P. (2017). The mechanism of sirtuin 2-mediated exacerbation of alpha-synuclein toxicity in models of Parkinson disease. PLoS Biol..

[157] Hassanzadeh K., Liu J., Maddila S., Mouradian M.M. (2024). Posttranslational Modifications of alpha-Synuclein, Their Therapeutic Potential, and Crosstalk in Health and Neurodegenerative Diseases. Pharmacol. Rev..

[158] Brown D.R. (2009). Metal binding to alpha-synuclein peptides and its contribution to toxicity. Biochem. Biophys. Res. Commun..

[159] Lorentzon E., Kumar R., Horvath I., Wittung-Stafshede P. (2020). Differential effects of Cu^2+^ and Fe^3+^ ions on in vitro amyloid formation of biologically-relevant alpha-synuclein variants. Biometals.

[160] Lothian A., Lago L., Mukherjee S., Connor A.R., Fowler C., McLean C.A., Horne M., Masters C.L., Cappai R., Roberts B.R. (2019). Characterization of the metal status of natively purified alpha-synuclein from human blood, brain tissue, or recombinant sources using size exclusion ICP-MS reveals no significant binding of Cu, Fe or Zn. Metallomics.

[161] Schmid A.W., Fauvet B., Moniatte M., Lashuel H.A. (2013). Alpha-synuclein post-translational modifications as potential biomarkers for Parkinson disease and other synucleinopathies. Mol. Cell. Proteom..

[162] Stephens A.D., Zacharopoulou M., Kaminski Schierle G.S. (2019). The Cellular Environment Affects Monomeric alpha-Synuclein Structure. Trends Biochem. Sci..

[163] Fowler D.M., Fields S. (2014). Deep mutational scanning: A new style of protein science. Nat. Methods.

[164] Newberry R.W., Leong J.T., Chow E.D., Kampmann M., DeGrado W.F. (2020). Deep mutational scanning reveals the structural basis for alpha-synuclein activity. Nat. Chem. Biol..

[165] Sherer T.B., Betarbet R., Stout A.K., Lund S., Baptista M., Panov A.V., Cookson M.R., Greenamyre J.T. (2002). An in vitro model of Parkinson’s disease: Linking mitochondrial impairment to altered alpha-synuclein metabolism and oxidative damage. J. Neurosci..

[166] Mishra S. (2023). Emerging Trends in Cryo-EM-based Structural Studies of Neuropathological Amyloids. J. Mol. Biol..

[167] Koga S., Sekiya H., Kondru N., Ross O.A., Dickson D.W. (2021). Neuropathology and molecular diagnosis of Synucleinopathies. Mol. Neurodegener..

[168] Todd T.W., Islam N.N., Cook C.N., Caulfield T.R., Petrucelli L. (2024). Cryo-EM structures of pathogenic fibrils and their impact on neurodegenerative disease research. Neuron.

[169] Roy S., Wolman L. (1969). Ultrastructural observations in Parkinsonism. J. Pathol..

[170] Tarutani A., Adachi T., Akatsu H., Hashizume Y., Hasegawa K., Saito Y., Robinson A.C., Mann D.M.A., Yoshida M., Murayama S. (2022). Ultrastructural and biochemical classification of pathogenic tau, alpha-synuclein and TDP-43. Acta Neuropathol..

[171] Kuzuhara S., Mori H., Izumiyama N., Yoshimura M., Ihara Y. (1988). Lewy bodies are ubiquitinated. A light and electron microscopic immunocytochemical study. Acta Neuropathol..

[172] Lowe J., Blanchard A., Morrell K., Lennox G., Reynolds L., Billett M., Landon M., Mayer R.J. (1988). Ubiquitin is a common factor in intermediate filament inclusion bodies of diverse type in man, including those of Parkinson’s disease, Pick’s disease, and Alzheimer’s disease, as well as Rosenthal fibres in cerebellar astrocytomas, cytoplasmic bodies in muscle, and mallory bodies in alcoholic liver disease. J. Pathol..

[173] Anderson J.P., Walker D.E., Goldstein J.M., de Laat R., Banducci K., Caccavello R.J., Barbour R., Huang J., Kling K., Lee M. (2006). Phosphorylation of Ser-129 is the dominant pathological modification of alpha-synuclein in familial and sporadic Lewy body disease. J. Biol. Chem..

[174] Wakabayashi K., Engelender S., Yoshimoto M., Tsuji S., Ross C.A., Takahashi H. (2000). Synphilin-1 is present in Lewy bodies in Parkinson’s disease. Ann. Neurol..

[175] Schlossmacher M.G., Frosch M.P., Gai W.P., Medina M., Sharma N., Forno L., Ochiishi T., Shimura H., Sharon R., Hattori N. (2002). Parkin localizes to the Lewy bodies of Parkinson disease and dementia with Lewy bodies. Am. J. Pathol..

[176] Ishizawa T., Mattila P., Davies P., Wang D., Dickson D.W. (2003). Colocalization of tau and alpha-synuclein epitopes in Lewy bodies. J. Neuropathol. Exp. Neurol..

[177] Zhu X., Babar A., Siedlak S.L., Yang Q., Ito G., Iwatsubo T., Smith M.A., Perry G., Chen S.G. (2006). LRRK2 in Parkinson’s disease and dementia with Lewy bodies. Mol. Neurodegener..

[178] Wakabayashi K., Tanji K., Mori F., Takahashi H. (2007). The Lewy body in Parkinson’s disease: Molecules implicated in the formation and degradation of alpha-synuclein aggregates. Neuropathology.

[179] Galvin J.E. (2024). Lewy Body Dementia. Contin. Lifelong Learn. Neurol..

[180] Braak H., Ghebremedhin E., Rub U., Bratzke H., Del Tredici K. (2004). Stages in the development of Parkinson’s disease-related pathology. Cell Tissue Res..

[181] Halliday G.M., Li Y.W., Blumbergs P.C., Joh T.H., Cotton R.G., Howe P.R., Blessing W.W., Geffen L.B. (1990). Neuropathology of immunohistochemically identified brainstem neurons in Parkinson’s disease. Ann. Neurol..

[182] Bernstein H.G., Johnson M., Perry R.H., LeBeau F.E., Dobrowolny H., Bogerts B., Perry E.K. (2011). Partial loss of parvalbumin-containing hippocampal interneurons in dementia with Lewy bodies. Neuropathology.

[183] Dugger B.N., Dickson D.W. (2010). Cell type specific sequestration of choline acetyltransferase and tyrosine hydroxylase within Lewy bodies. Acta Neuropathol..

[184] Marui W., Iseki E., Kato M., Kosaka K. (2003). Degeneration of tyrosine hydroxylase-immunoreactive neurons in the cerebral cortex and hippocampus of patients with dementia with Lewy bodies. Neurosci. Lett..

[185] Beach T.G., Adler C.H., Lue L., Sue L.I., Bachalakuri J., Henry-Watson J., Sasse J., Boyer S., Shirohi S., Brooks R. (2009). Unified staging system for Lewy body disorders: Correlation with nigrostriatal degeneration, cognitive impairment and motor dysfunction. Acta Neuropathol..

[186] Harding A.J., Stimson E., Henderson J.M., Halliday G.M. (2002). Clinical correlates of selective pathology in the amygdala of patients with Parkinson’s disease. Brain.

[187] Mattila P.M., Rinne J.O., Helenius H., Dickson D.W., Roytta M. (2000). Alpha-synuclein-immunoreactive cortical Lewy bodies are associated with cognitive impairment in Parkinson’s disease. Acta Neuropathol..

[188] Kramer M.L., Schulz-Schaeffer W.J. (2007). Presynaptic alpha-synuclein aggregates, not Lewy bodies, cause neurodegeneration in dementia with Lewy bodies. J. Neurosci..

[189] Kalia L.V., Lang A.E., Hazrati L.N., Fujioka S., Wszolek Z.K., Dickson D.W., Ross O.A., Van Deerlin V.M., Trojanowski J.Q., Hurtig H.I. (2015). Clinical correlations with Lewy body pathology in LRRK2-related Parkinson disease. JAMA Neurol..

[190] Mahul-Mellier A.L., Burtscher J., Maharjan N., Weerens L., Croisier M., Kuttler F., Leleu M., Knott G.W., Lashuel H.A. (2020). The process of Lewy body formation, rather than simply alpha-synuclein fibrillization, is one of the major drivers of neurodegeneration. Proc. Natl. Acad. Sci. USA.

[191] Betzer C., Lassen L.B., Olsen A., Kofoed R.H., Reimer L., Gregersen E., Zheng J., Cali T., Gai W.P., Chen T. (2018). Alpha-synuclein aggregates activate calcium pump SERCA leading to calcium dysregulation. EMBO Rep..

[192] Moussaud S., Jones D.R., Moussaud-Lamodiere E.L., Delenclos M., Ross O.A., McLean P.J. (2014). Alpha-synuclein and tau: Teammates in neurodegeneration?. Mol. Neurodegener..

[193] Schaefer A., Naser D., Siebeneichler B., Tarasca M.V., Meiering E.M. (2022). Methodological advances and strategies for high resolution structure determination of cellular protein aggregates. J. Biol. Chem..

[194] Altay M.F., Kumar S.T., Burtscher J., Jagannath S., Strand C., Miki Y., Parkkinen L., Holton J.L., Lashuel H.A. (2023). Development and validation of an expanded antibody toolset that captures alpha-synuclein pathological diversity in Lewy body diseases. NPJ Park. Dis..

[195] Verdurand M., Levigoureux E., Zeinyeh W., Berthier L., Mendjel-Herda M., Cadarossanesaib F., Bouillot C., Iecker T., Terreux R., Lancelot S. (2018). In Silico, In Vitro, and In Vivo Evaluation of New Candidates for Alpha-Synuclein PET Imaging. Mol. Pharm..

[196] Orlovskaya V.V., Fedorova O.S., Viktorov N.B., Vaulina D.D., Krasikova R.N. (2023). One-Pot Radiosynthesis of [^18^F]Anle138b-5-(3-Bromophenyl)-3-(6-[^18^F]fluorobenzo[d][1,3]dioxol-5-yl)-1H-pyrazole-A Potential PET Radiotracer Targeting alpha-Synuclein Aggregates. Molecules.

[197] Koh Y.H., Tan L.Y., Ng S.Y. (2018). Patient-Derived Induced Pluripotent Stem Cells and Organoids for Modeling Alpha Synuclein Propagation in Parkinson’s Disease. Front. Cell. Neurosci..

[198] Van der Perren A., Gelders G., Fenyi A., Bousset L., Brito F., Peelaerts W., Van den Haute C., Gentleman S., Melki R., Baekelandt V. (2020). The structural differences between patient-derived alpha-synuclein strains dictate characteristics of Parkinson’s disease, multiple system atrophy and dementia with Lewy bodies. Acta Neuropathol..

[199] Schmitz M., Candelise N., Canaslan S., Altmeppen H.C., Matschke J., Glatzel M., Younas N., Zafar S., Hermann P., Zerr I. (2023). Alpha-Synuclein conformers reveal link to clinical heterogeneity of alpha-synucleinopathies. Transl. Neurodegener..

[200] Kline E.M., Houser M.C., Herrick M.K., Seibler P., Klein C., West A., Tansey M.G. (2021). Genetic and Environmental Factors in Parkinson’s Disease Converge on Immune Function and Inflammation. Mov. Disord..

[201] Calabresi P., Mechelli A., Natale G., Volpicelli-Daley L., Di Lazzaro G., Ghiglieri V. (2023). Alpha-synuclein in Parkinson’s disease and other synucleinopathies: From overt neurodegeneration back to early synaptic dysfunction. Cell Death Dis..

[202] Xu W., Tan L., Yu J.T. (2015). Link between the SNCA gene and parkinsonism. Neurobiol. Aging.

[203] Blauwendraat C., Nalls M.A., Singleton A.B. (2020). The genetic architecture of Parkinson’s disease. Lancet Neurol..

[204] Orme T., Guerreiro R., Bras J. (2018). The Genetics of Dementia with Lewy Bodies: Current Understanding and Future Directions. Curr. Neurol. Neurosci. Rep..

[205] Katzeff J.S., Phan K., Purushothuman S., Halliday G.M., Kim W.S. (2019). Cross-examining candidate genes implicated in multiple system atrophy. Acta Neuropathol. Commun..

[206] Bardien S., Lesage S., Brice A., Carr J. (2011). Genetic characteristics of leucine-rich repeat kinase 2 (LRRK2) associated Parkinson’s disease. Park. Relat. Disord..

[207] Dos Santos J.C.C., Mano G.B.C., da Cunha Barreto-Vianna A.R., Garcia T.F.M., de Vasconcelos A.V., Sa C.S.G., de Souza Santana S.L., Farias A.G.P., Seimaru B., Lima M.P.P. (2024). The Molecular Impact of Glucosylceramidase Beta 1 (Gba1) in Parkinson’s Disease: A New Genetic State of the Art. Mol. Neurobiol..

[208] Granek Z., Barczuk J., Siwecka N., Rozpedek-Kaminska W., Kucharska E., Majsterek I. (2023). GBA1 Gene Mutations in alpha-Synucleinopathies-Molecular Mechanisms Underlying Pathology and Their Clinical Significance. Int. J. Mol. Sci..

[209] Lwin A., Orvisky E., Goker-Alpan O., LaMarca M.E., Sidransky E. (2004). Glucocerebrosidase mutations in subjects with parkinsonism. Mol. Genet. Metab..

[210] Sidransky E., Nalls M.A., Aasly J.O., Aharon-Peretz J., Annesi G., Barbosa E.R., Bar-Shira A., Berg D., Bras J., Brice A. (2009). Multicenter analysis of glucocerebrosidase mutations in Parkinson’s disease. N. Engl. J. Med..

[211] Nussbaum R.L. (2018). Genetics of Synucleinopathies. Cold Spring Harb. Perspect. Med..

[212] Chopra A., Outeiro T.F. (2024). Aggregation and beyond: Alpha-synuclein-based biomarkers in synucleinopathies. Brain.

[213] Chiba-Falek O. (2017). Structural variants in SNCA gene and the implication to synucleinopathies. Curr. Opin. Genet. Dev..

[214] Book A., Guella I., Candido T., Brice A., Hattori N., Jeon B., Farrer M.J., SNCA Multiplication Investigators of the GEoPD Consortium (2018). A Meta-Analysis of alpha-Synuclein Multiplication in Familial Parkinsonism. Front. Neurol..

[215] Tsalenchuk M., Gentleman S.M., Marzi S.J. (2023). Linking environmental risk factors with epigenetic mechanisms in Parkinson’s disease. NPJ Park. Dis..

[216] Guhathakurta S., Bok E., Evangelista B.A., Kim Y.S. (2017). Deregulation of alpha-synuclein in Parkinson’s disease: Insight from epigenetic structure and transcriptional regulation of SNCA. Prog. Neurobiol..

[217] Miranda-Morales E., Meier K., Sandoval-Carrillo A., Salas-Pacheco J., Vazquez-Cardenas P., Arias-Carrion O. (2017). Implications of DNA Methylation in Parkinson’s Disease. Front. Mol. Neurosci..

[218] Hatano T., Okuzumi A., Matsumoto G., Tsunemi T., Hattori N. (2024). Alpha-Synuclein: A Promising Biomarker for Parkinson’s Disease and Related Disorders. J. Mov. Disord..

[219] Malfertheiner K., Stefanova N., Heras-Garvin A. (2021). The Concept of alpha-Synuclein Strains and How Different Conformations May Explain Distinct Neurodegenerative Disorders. Front. Neurol..

[220] Smith L.J., Lee C.Y., Menozzi E., Schapira A.H.V. (2022). Genetic variations in GBA1 and LRRK2 genes: Biochemical and clinical consequences in Parkinson disease. Front. Neurol..

[221] Redensek S., Dolzan V., Kunej T. (2018). From Genomics to Omics Landscapes of Parkinson’s Disease: Revealing the Molecular Mechanisms. Omics J. Integr. Biol..

[222] Kordower J.H., Chu Y., Hauser R.A., Freeman T.B., Olanow C.W. (2008). Lewy body-like pathology in long-term embryonic nigral transplants in Parkinson’s disease. Nat. Med..

[223] Killinger B.A., Kordower J.H. (2019). Spreading of alpha-synuclein—Relevant or epiphenomenon?. J. Neurochem..

[224] Bras I.C., Outeiro T.F. (2021). Alpha-Synuclein: Mechanisms of Release and Pathology Progression in Synucleinopathies. Cells.

[225] Hijaz B.A., Volpicelli-Daley L.A. (2020). Initiation and propagation of alpha-synuclein aggregation in the nervous system. Mol. Neurodegener..

[226] Grozdanov V., Danzer K.M. (2018). Release and uptake of pathologic alpha-synuclein. Cell Tissue Res..

[227] Holmes B.B., DeVos S.L., Kfoury N., Li M., Jacks R., Yanamandra K., Ouidja M.O., Brodsky F.M., Marasa J., Bagchi D.P. (2013). Heparan sulfate proteoglycans mediate internalization and propagation of specific proteopathic seeds. Proc. Natl. Acad. Sci. USA.

[228] Rodriguez L., Marano M.M., Tandon A. (2018). Import and Export of Misfolded alpha-Synuclein. Front. Neurosci..

[229] Domingues R., Sant’Anna R., da Fonseca A.C.C., Robbs B.K., Foguel D., Outeiro T.F. (2022). Extracellular alpha-synuclein: Sensors, receptors, and responses. Neurobiol. Dis..

[230] Xiong M., Xia D., Yu H., Meng L., Zhang X., Chen J., Tian Y., Yuan X., Niu X., Nie S. (2024). Microglia Process alpha-Synuclein Fibrils and Enhance their Pathogenicity in a TREM2-Dependent Manner. Adv. Sci..

[231] Uemura N., Ueda J., Yoshihara T., Ikuno M., Uemura M.T., Yamakado H., Asano M., Trojanowski J.Q., Takahashi R. (2021). Alpha-Synuclein Spread from Olfactory Bulb Causes Hyposmia, Anxiety, and Memory Loss in BAC-SNCA Mice. Mov. Disord..

[232] Yoo H., Lee J., Kim B., Moon H., Jeong H., Lee K., Song W.J., Hur J.K., Oh Y. (2022). Role of post-translational modifications on the alpha-synuclein aggregation-related pathogenesis of Parkinson’s disease. BMB Rep..

[233] Lashuel H.A., Mahul-Mellier A.L., Novello S., Hegde R.N., Jasiqi Y., Altay M.F., Donzelli S., DeGuire S.M., Burai R., Magalhaes P. (2022). Revisiting the specificity and ability of phospho-S129 antibodies to capture alpha-synuclein biochemical and pathological diversity. NPJ Park. Dis..

[234] Angot E., Steiner J.A., Hansen C., Li J.Y., Brundin P. (2010). Are synucleinopathies prion-like disorders?. Lancet Neurol..

[235] Zampar S., Di Gregorio S.E., Grimmer G., Watts J.C., Ingelsson M. (2024). “Prion-like” seeding and propagation of oligomeric protein assemblies in neurodegenerative disorders. Front. Neurosci..

[236] Koprich J.B., Kalia L.V., Brotchie J.M. (2017). Animal models of alpha-synucleinopathy for Parkinson disease drug development. Nat. Rev. Neurosci..

[237] Dehay B., Fernagut P.O. (2016). Alpha-synuclein-based models of Parkinson’s disease. Rev. Neurol..

[238] Doorn K.J., Moors T., Drukarch B., van de Berg W., Lucassen P.J., van Dam A.M. (2014). Microglial phenotypes and toll-like receptor 2 in the substantia nigra and hippocampus of incidental Lewy body disease cases and Parkinson’s disease patients. Acta Neuropathol. Commun..

[239] Iannaccone S., Cerami C., Alessio M., Garibotto V., Panzacchi A., Olivieri S., Gelsomino G., Moresco R.M., Perani D. (2013). In vivo microglia activation in very early dementia with Lewy bodies, comparison with Parkinson’s disease. Park. Relat. Disord..

[240] Imamura K., Hishikawa N., Sawada M., Nagatsu T., Yoshida M., Hashizume Y. (2003). Distribution of major histocompatibility complex class II-positive microglia and cytokine profile of Parkinson’s disease brains. Acta Neuropathol..

[241] Ouchi Y., Yoshikawa E., Sekine Y., Futatsubashi M., Kanno T., Ogusu T., Torizuka T. (2005). Microglial activation and dopamine terminal loss in early Parkinson’s disease. Ann. Neurol..

[242] Lv Q.K., Tao K.X., Wang X.B., Yao X.Y., Pang M.Z., Liu J.Y., Wang F., Liu C.F. (2023). Role of alpha-synuclein in microglia: Autophagy and phagocytosis balance neuroinflammation in Parkinson’s disease. Inflamm. Res..

[243] Hoffmann A., Ettle B., Bruno A., Kulinich A., Hoffmann A.C., von Wittgenstein J., Winkler J., Xiang W., Schlachetzki J.C.M. (2016). Alpha-synuclein activates BV2 microglia dependent on its aggregation state. Biochem. Biophys. Res. Commun..

[244] Huang Q., Yang P., Liu Y., Ding J., Lu M., Hu G. (2023). The interplay between alpha-Synuclein and NLRP3 inflammasome in Parkinson’s disease. Biomed. Pharmacother..

[245] Demirtas N., Mazlumoglu B.S., Palabiyik Yucelik S.S. (2023). Role of NLRP3 Inflammasomes in Neurodegenerative Diseases. Eurasian J. Med..

[246] Wang Q., Liu Y., Zhou J. (2015). Neuroinflammation in Parkinson’s disease and its potential as therapeutic target. Transl. Neurodegener..

[247] Yildirim-Balatan C., Fenyi A., Besnault P., Gomez L., Sepulveda-Diaz J.E., Michel P.P., Melki R., Hunot S. (2024). Parkinson’s disease-derived alpha-synuclein assemblies combined with chronic-type inflammatory cues promote a neurotoxic microglial phenotype. J. Neuroinflamm..

[248] Delgado-Minjares K.M., Martinez-Fong D., Martinez-Davila I.A., Banuelos C., Gutierrez-Castillo M.E., Blanco-Alvarez V.M., Cardenas-Aguayo M.D., Luna-Munoz J., Pacheco-Herrero M., Soto-Rojas L.O. (2021). Mechanistic Insight from Preclinical Models of Parkinson’s Disease Could Help Redirect Clinical Trial Efforts in GDNF Therapy. Int. J. Mol. Sci..

[249] Heintz-Buschart A., Pandey U., Wicke T., Sixel-Doring F., Janzen A., Sittig-Wiegand E., Trenkwalder C., Oertel W.H., Mollenhauer B., Wilmes P. (2018). The nasal and gut microbiome in Parkinson’s disease and idiopathic rapid eye movement sleep behavior disorder. Mov. Disord..

[250] Hill-Burns E.M., Debelius J.W., Morton J.T., Wissemann W.T., Lewis M.R., Wallen Z.D., Peddada S.D., Factor S.A., Molho E., Zabetian C.P. (2017). Parkinson’s disease and Parkinson’s disease medications have distinct signatures of the gut microbiome. Mov. Disord..

[251] Keshavarzian A., Green S.J., Engen P.A., Voigt R.M., Naqib A., Forsyth C.B., Mutlu E., Shannon K.M. (2015). Colonic bacterial composition in Parkinson’s disease. Mov. Disord..

[252] Kleine Bardenhorst S., Cereda E., Severgnini M., Barichella M., Pezzoli G., Keshavarzian A., Desideri A., Pietrucci D., Aho V.T.E., Scheperjans F. (2023). Gut microbiota dysbiosis in Parkinson disease: A systematic review and pooled analysis. Eur. J. Neurol..

[253] Nie S., Ge Y. (2023). The link between the gut microbiome, inflammation, and Parkinson’s disease. Appl. Microbiol. Biotechnol..

[254] Chen S.G., Stribinskis V., Rane M.J., Demuth D.R., Gozal E., Roberts A.M., Jagadapillai R., Liu R., Choe K., Shivakumar B. (2016). Exposure to the Functional Bacterial Amyloid Protein Curli Enhances Alpha-Synuclein Aggregation in Aged Fischer 344 Rats and Caenorhabditis elegans. Sci. Rep..

[255] Alam M., Abbas K., Mustafa M., Usmani N., Habib S. (2024). Microbiome-based therapies for Parkinson’s disease. Front. Nutr..

[256] Braak H., Del Tredici K., Rub U., de Vos R.A., Jansen Steur E.N., Braak E. (2003). Staging of brain pathology related to sporadic Parkinson’s disease. Neurobiol. Aging.

[257] Consonni A., Miglietti M., De Luca C.M.G., Cazzaniga F.A., Ciullini A., Dellarole I.L., Bufano G., Di Fonzo A., Giaccone G., Baggi F. (2022). Approaching the Gut and Nasal Microbiota in Parkinson’s Disease in the Era of the Seed Amplification Assays. Brain Sci..

[258] Cristaldi A., Fiore M., Oliveri Conti G., Pulvirenti E., Favara C., Grasso A., Copat C., Ferrante M. (2022). Possible association between PM(2.5) and neurodegenerative diseases: A systematic review. Environ. Res..

[259] Barrenschee M., Zorenkov D., Bottner M., Lange C., Cossais F., Scharf A.B., Deuschl G., Schneider S.A., Ellrichmann M., Fritscher-Ravens A. (2017). Distinct pattern of enteric phospho-alpha-synuclein aggregates and gene expression profiles in patients with Parkinson’s disease. Acta Neuropathol. Commun..

[260] Casini A., Vivacqua G., Ceci L., Leone S., Vaccaro R., Tagliafierro M., Bassi F.M., Vitale S., Bocci E., Pannarale L. (2024). TNBS colitis induces architectural changes and alpha-synuclein overexpression in mouse distal colon: A morphological study. Cell Tissue Res..

[261] Holmqvist S., Chutna O., Bousset L., Aldrin-Kirk P., Li W., Bjorklund T., Wang Z.Y., Roybon L., Melki R., Li J.Y. (2014). Direct evidence of Parkinson pathology spread from the gastrointestinal tract to the brain in rats. Acta Neuropathol..

[262] Kim J.Y., Illigens B.M., McCormick M.P., Wang N., Gibbons C.H. (2019). Alpha-Synuclein in Skin Nerve Fibers as a Biomarker for Alpha-Synucleinopathies. J. Clin. Neurol..

[263] Carabotti M., Scirocco A., Maselli M.A., Severi C. (2015). The gut-brain axis: Interactions between enteric microbiota, central and enteric nervous systems. Ann. Gastroenterol..

[264] Duan W.X., Wang F., Liu J.Y., Liu C.F. (2024). Relationship Between Short-chain Fatty Acids and Parkinson’s Disease: A Review from Pathology to Clinic. Neurosci. Bull..

[265] Killinger B.A., Madaj Z., Sikora J.W., Rey N., Haas A.J., Vepa Y., Lindqvist D., Chen H., Thomas P.M., Brundin P. (2018). The vermiform appendix impacts the risk of developing Parkinson’s disease. Sci. Transl. Med..

[266] Mendes A., Goncalves A., Vila-Cha N., Moreira I., Fernandes J., Damasio J., Teixeira-Pinto A., Taipa R., Lima A.B., Cavaco S. (2015). Appendectomy may delay Parkinson’s disease Onset. Mov. Disord..

[267] Svensson E., Horvath-Puho E., Thomsen R.W., Djurhuus J.C., Pedersen L., Borghammer P., Sorensen H.T. (2015). Vagotomy and subsequent risk of Parkinson’s disease. Ann. Neurol..

[268] Park S.J., Kim K.W., Lee E.J. (2024). Gut-brain axis and environmental factors in Parkinson’s disease: Bidirectional link between disease onset and progression. Neural Regen. Res..

[269] Jo J., Yang L., Tran H.D., Yu W., Sun A.X., Chang Y.Y., Jung B.C., Lee S.J., Saw T.Y., Xiao B. (2021). Lewy Body-like Inclusions in Human Midbrain Organoids Carrying Glucocerebrosidase and alpha-Synuclein Mutations. Ann. Neurol..

[270] Yang Y., Shi Y., Schweighauser M., Zhang X., Kotecha A., Murzin A.G., Garringer H.J., Cullinane P.W., Saito Y., Foroud T. (2022). Structures of alpha-synuclein filaments from human brains with Lewy pathology. Nature.

[271] Giraldez-Perez R., Antolin-Vallespin M., Munoz M., Sanchez-Capelo A. (2014). Models of alpha-synuclein aggregation in Parkinson’s disease. Acta Neuropathol. Commun..

[272] Dovonou A., Bolduc C., Soto Linan V., Gora C., Peralta Iii M.R., Levesque M. (2023). Animal models of Parkinson’s disease: Bridging the gap between disease hallmarks and research questions. Transl. Neurodegener..

[273] Feany M.B., Bender W.W. (2000). A Drosophila model of Parkinson’s disease. Nature.

[274] Kahle P.J., Neumann M., Ozmen L., Muller V., Jacobsen H., Schindzielorz A., Okochi M., Leimer U., van Der Putten H., Probst A. (2000). Subcellular localization of wild-type and Parkinson’s disease-associated mutant alpha -synuclein in human and transgenic mouse brain. J. Neurosci..

[275] Masliah E., Rockenstein E., Veinbergs I., Mallory M., Hashimoto M., Takeda A., Sagara Y., Sisk A., Mucke L. (2000). Dopaminergic loss and inclusion body formation in alpha-synuclein mice: Implications for neurodegenerative disorders. Science.

[276] Olsen A.L., Feany M.B. (2019). Glial alpha-synuclein promotes neurodegeneration characterized by a distinct transcriptional program in vivo. Glia.

[277] Gomez-Benito M., Granado N., Garcia-Sanz P., Michel A., Dumoulin M., Moratalla R. (2020). Modeling Parkinson’s Disease With the Alpha-Synuclein Protein. Front. Pharmacol..

[278] Ulusoy A., Decressac M., Kirik D., Bjorklund A. (2010). Viral vector-mediated overexpression of alpha-synuclein as a progressive model of Parkinson’s disease. Prog. Brain Res..

[279] Bjorklund A., Mattsson B. (2024). The AAV-alpha-Synuclein Model of Parkinson’s Disease: An Update. J. Park. Dis..

[280] Chung H.K., Ho H.A., Perez-Acuna D., Lee S.J. (2020). Modeling alpha-Synuclein Propagation with Preformed Fibril Injections. J. Mov. Disord..

[281] Akkentli F., Jang I.K., Choi Y., Min Y., Park J., Jo H., Kim L., Mendpara A., Bains B., Yoo D. (2024). Quantitative proteomic analysis using a mouse model of Lewy body dementia induced by alpha-synuclein preformed fibrils injection. Front. Dement..

[282] Luk K.C., Kehm V.M., Zhang B., O’Brien P., Trojanowski J.Q., Lee V.M. (2012). Intracerebral inoculation of pathological alpha-synuclein initiates a rapidly progressive neurodegenerative alpha-synucleinopathy in mice. J. Exp. Med..

[283] Luk K.C., Song C., O’Brien P., Stieber A., Branch J.R., Brunden K.R., Trojanowski J.Q., Lee V.M. (2009). Exogenous alpha-synuclein fibrils seed the formation of Lewy body-like intracellular inclusions in cultured cells. Proc. Natl. Acad. Sci. USA.

[284] Dadgar-Kiani E., Bieri G., Melki R., Hossain A., Gitler A.D., Lee J.H. (2024). Neuromodulation modifies alpha-synuclein spreading dynamics in vivo and the pattern is predicted by changes in whole-brain function. Brain Stimul..

[285] Hofs L., Geissler-Losch D., Wunderlich K.M., Szego E.M., Van den Haute C., Baekelandt V., Hoyer W., Falkenburger B.H. (2024). Evaluation of the Effect of beta-Wrapin AS69 in a Mouse Model Based on Alpha-Synuclein Overexpression. Biomolecules.

[286] Challis C., Hori A., Sampson T.R., Yoo B.B., Challis R.C., Hamilton A.M., Mazmanian S.K., Volpicelli-Daley L.A., Gradinaru V. (2020). Gut-seeded alpha-synuclein fibrils promote gut dysfunction and brain pathology specifically in aged mice. Nat. Neurosci..

[287] Delenclos M., Burgess J.D., Lamprokostopoulou A., Outeiro T.F., Vekrellis K., McLean P.J. (2019). Cellular models of alpha-synuclein toxicity and aggregation. J. Neurochem..

[288] Surmeier D.J., Obeso J.A., Halliday G.M. (2017). Selective neuronal vulnerability in Parkinson disease. Nat. Rev. Neurosci..

[289] Zeng X.S., Geng W.S., Jia J.J. (2018). Neurotoxin-Induced Animal Models of Parkinson Disease: Pathogenic Mechanism and Assessment. ASN Neuro.

[290] Luna-Herrera C., Martinez-Davila I.A., Soto-Rojas L.O., Flores-Martinez Y.M., Fernandez-Parrilla M.A., Ayala-Davila J., Leon-Chavez B.A., Soto-Rodriguez G., Blanco-Alvarez V.M., Lopez-Salas F.E. (2020). Intranigral Administration of beta-Sitosterol-beta-D-Glucoside Elicits Neurotoxic A1 Astrocyte Reactivity and Chronic Neuroinflammation in the Rat Substantia Nigra. J. Immunol. Res..

[291] Morales-Martinez A., Martinez-Gomez P.A., Martinez-Fong D., Villegas-Rojas M.M., Perez-Severiano F., Del Toro-Colin M.A., Delgado-Minjares K.M., Blanco-Alvarez V.M., Leon-Chavez B.A., Aparicio-Trejo O.E. (2022). Oxidative Stress and Mitochondrial Complex I Dysfunction Correlate with Neurodegeneration in an alpha-Synucleinopathy Animal Model. Int. J. Mol. Sci..

[292] Walsh N.C., Kenney L.L., Jangalwe S., Aryee K.E., Greiner D.L., Brehm M.A., Shultz L.D. (2017). Humanized Mouse Models of Clinical Disease. Annu. Rev. Pathol..

[293] Gamache J., Benzow K., Forster C., Kemper L., Hlynialuk C., Furrow E., Ashe K.H., Koob M.D. (2019). Factors other than hTau overexpression that contribute to tauopathy-like phenotype in rTg4510 mice. Nat. Commun..

[294] Kweon S.H., Ryu H.G., Kwon S.H., Park H., Lee S., Kim N.S., Ma S.X., Jee H.J., Kim S., Ko H.S. (2024). Gba1 E326K renders motor and non-motor symptoms with pathological alpha-synuclein, tau and glial activation. Brain.

[295] Kim Y., McInnes J., Kim J., Liang Y.H.W., Veeraragavan S., Garza A.R., Belfort B.D.W., Arenkiel B., Samaco R., Zoghbi H.Y. (2024). Olfactory deficit and gastrointestinal dysfunction precede motor abnormalities in alpha-Synuclein G51D knock-in mice. Proc. Natl. Acad. Sci. USA.

[296] Teil M., Arotcarena M.L., Dehay B. (2021). A New Rise of Non-Human Primate Models of Synucleinopathies. Biomedicines.

[297] Marvian A.T., Koss D.J., Aliakbari F., Morshedi D., Outeiro T.F. (2019). In vitro models of synucleinopathies: Informing on molecular mechanisms and protective strategies. J. Neurochem..

[298] Lazaro D.F., Pavlou M.A.S., Outeiro T.F. (2017). Cellular models as tools for the study of the role of alpha-synuclein in Parkinson’s disease. Exp. Neurol..

[299] Vasili E., Dominguez-Meijide A., Outeiro T.F. (2019). Spreading of alpha-Synuclein and Tau: A Systematic Comparison of the Mechanisms Involved. Front. Mol. Neurosci..

[300] Cenci M.A., Bjorklund A. (2020). Animal models for preclinical Parkinson’s research: An update and critical appraisal. Prog. Brain Res..

[301] Lee H.J., Shin S.Y., Choi C., Lee Y.H., Lee S.J. (2002). Formation and removal of alpha-synuclein aggregates in cells exposed to mitochondrial inhibitors. J. Biol. Chem..

[302] McLean P.J., Kawamata H., Hyman B.T. (2001). Alpha-synuclein-enhanced green fluorescent protein fusion proteins form proteasome sensitive inclusions in primary neurons. Neuroscience.

[303] Volpicelli-Daley L.A., Gamble K.L., Schultheiss C.E., Riddle D.M., West A.B., Lee V.M. (2014). Formation of alpha-synuclein Lewy neurite-like aggregates in axons impedes the transport of distinct endosomes. Mol. Biol. Cell.

[304] Klucken J., Poehler A.M., Ebrahimi-Fakhari D., Schneider J., Nuber S., Rockenstein E., Schlotzer-Schrehardt U., Hyman B.T., McLean P.J., Masliah E. (2012). Alpha-synuclein aggregation involves a bafilomycin A 1-sensitive autophagy pathway. Autophagy.

[305] Lazaro D.F., Rodrigues E.F., Langohr R., Shahpasandzadeh H., Ribeiro T., Guerreiro P., Gerhardt E., Krohnert K., Klucken J., Pereira M.D. (2014). Systematic comparison of the effects of alpha-synuclein mutations on its oligomerization and aggregation. PLoS Genet..

[306] Outeiro T.F., Putcha P., Tetzlaff J.E., Spoelgen R., Koker M., Carvalho F., Hyman B.T., McLean P.J. (2008). Formation of toxic oligomeric alpha-synuclein species in living cells. PLoS ONE.

[307] Kragh C.L., Lund L.B., Febbraro F., Hansen H.D., Gai W.P., El-Agnaf O., Richter-Landsberg C., Jensen P.H. (2009). Alpha-synuclein aggregation and Ser-129 phosphorylation-dependent cell death in oligodendroglial cells. J. Biol. Chem..

[308] Conway K.A., Lee S.J., Rochet J.C., Ding T.T., Harper J.D., Williamson R.E., Lansbury P.T. (2000). Accelerated oligomerization by Parkinson’s disease linked alpha-synuclein mutants. Ann. N. Y. Acad. Sci..

[309] Villar-Pique A., Lopes da Fonseca T., Sant’Anna R., Szego E.M., Fonseca-Ornelas L., Pinho R., Carija A., Gerhardt E., Masaracchia C., Abad Gonzalez E. (2016). Environmental and genetic factors support the dissociation between alpha-synuclein aggregation and toxicity. Proc. Natl. Acad. Sci. USA.

[310] Abati E., Di Fonzo A., Corti S. (2018). In vitro models of multiple system atrophy from primary cells to induced pluripotent stem cells. J. Cell. Mol. Med..

[311] Tanaka M., Kim Y.M., Lee G., Junn E., Iwatsubo T., Mouradian M.M. (2004). Aggresomes formed by alpha-synuclein and synphilin-1 are cytoprotective. J. Biol. Chem..

[312] Lindersson E., Beedholm R., Hojrup P., Moos T., Gai W., Hendil K.B., Jensen P.H. (2004). Proteasomal inhibition by alpha-synuclein filaments and oligomers. J. Biol. Chem..

[313] Danzer K.M., Kranich L.R., Ruf W.P., Cagsal-Getkin O., Winslow A.R., Zhu L., Vanderburg C.R., McLean P.J. (2012). Exosomal cell-to-cell transmission of alpha synuclein oligomers. Mol. Neurodegener..

[314] Jiang H., Tang M., Xu Z., Wang Y., Li M., Zheng S., Zhu J., Lin Z., Zhang M. (2024). CRISPR/Cas9 system and its applications in nervous system diseases. Genes Dis..

[315] Jankovic J., Tan E.K. (2020). Parkinson’s disease: Etiopathogenesis and treatment. J. Neurol. Neurosurg. Psychiatry.

[316] Pal G., Cook L., Schulze J., Verbrugge J., Alcalay R.N., Merello M., Sue C.M., Bardien S., Bonifati V., Chung S.J. (2023). Genetic Testing in Parkinson’s Disease. Mov. Disord..

[317] Maass F., Schulz I., Lingor P., Mollenhauer B., Bahr M. (2019). Cerebrospinal fluid biomarker for Parkinson’s disease: An overview. Mol. Cell. Neurosci..

[318] Eusebi P., Giannandrea D., Biscetti L., Abraha I., Chiasserini D., Orso M., Calabresi P., Parnetti L. (2017). Diagnostic utility of cerebrospinal fluid alpha-synuclein in Parkinson’s disease: A systematic review and meta-analysis. Mov. Disord..

[319] Fayyad M., Salim S., Majbour N., Erskine D., Stoops E., Mollenhauer B., El-Agnaf O.M.A. (2019). Parkinson’s disease biomarkers based on alpha-synuclein. J. Neurochem..

[320] Hansson O., Hall S., Ohrfelt A., Zetterberg H., Blennow K., Minthon L., Nagga K., Londos E., Varghese S., Majbour N.K. (2014). Levels of cerebrospinal fluid alpha-synuclein oligomers are increased in Parkinson’s disease with dementia and dementia with Lewy bodies compared to Alzheimer’s disease. Alzheimers Res. Ther..

[321] Parnetti L., Chiasserini D., Persichetti E., Eusebi P., Varghese S., Qureshi M.M., Dardis A., Deganuto M., De Carlo C., Castrioto A. (2014). Cerebrospinal fluid lysosomal enzymes and alpha-synuclein in Parkinson’s disease. Mov. Disord..

[322] Tokuda T., Salem S.A., Allsop D., Mizuno T., Nakagawa M., Qureshi M.M., Locascio J.J., Schlossmacher M.G., El-Agnaf O.M. (2006). Decreased alpha-synuclein in cerebrospinal fluid of aged individuals and subjects with Parkinson’s disease. Biochem. Biophys. Res. Commun..

[323] Porro C., Panaro M.A., Lofrumento D.D., Hasalla E., Trotta T. (2019). The multiple roles of exosomes in Parkinson’s disease: An overview. Immunopharmacol. Immunotoxicol..

[324] Mollenhauer B., Caspell-Garcia C.J., Coffey C.S., Taylor P., Singleton A., Shaw L.M., Trojanowski J.Q., Frasier M., Simuni T., Iranzo A. (2019). Longitudinal analyses of cerebrospinal fluid alpha-Synuclein in prodromal and early Parkinson’s disease. Mov. Disord..

[325] Cairns A.G., Vazquez-Romero A., Mahdi Moein M., Aden J., Elmore C.S., Takano A., Arakawa R., Varrone A., Almqvist F., Schou M. (2018). Increased Brain Exposure of an Alpha-Synuclein Fibrillization Modulator by Utilization of an Activated Ester Prodrug Strategy. ACS Chem. Neurosci..

[326] Merchant K.M., Cedarbaum J.M., Brundin P., Dave K.D., Eberling J., Espay A.J., Hutten S.J., Javidnia M., Luthman J., Maetzler W. (2019). A Proposed Roadmap for Parkinson’s Disease Proof of Concept Clinical Trials Investigating Compounds Targeting Alpha-Synuclein. J. Park. Dis..

[327] Manne S., Kondru N., Hepker M., Jin H., Anantharam V., Lewis M., Huang X., Kanthasamy A., Kanthasamy A.G. (2019). Ultrasensitive Detection of Aggregated alpha-Synuclein in Glial Cells, Human Cerebrospinal Fluid, and Brain Tissue Using the RT-QuIC Assay: New High-Throughput Neuroimmune Biomarker Assay for Parkinsonian Disorders. J. Neuroimmune Pharmacol..

[328] Saijo E., Groveman B.R., Kraus A., Metrick M., Orru C.D., Hughson A.G., Caughey B. (2019). Ultrasensitive RT-QuIC Seed Amplification Assays for Disease-Associated Tau, alpha-Synuclein, and Prion Aggregates. Methods Mol. Biol..

[329] Vaikath N.N., Hmila I., Gupta V., Erskine D., Ingelsson M., El-Agnaf O.M.A. (2019). Antibodies against alpha-synuclein: Tools and therapies. J. Neurochem..

[330] Hollerhage M., Wolff A., Chakroun T., Evsyukov V., Duan L., Chua O.W., Tang Q., Koeglsperger T., Hoglinger G.U. (2022). Binding Stability of Antibody-alpha-Synuclein Complexes Predicts the Protective Efficacy of Anti-alpha-synuclein Antibodies. Mol. Neurobiol..

[331] Covell D.J., Robinson J.L., Akhtar R.S., Grossman M., Weintraub D., Bucklin H.M., Pitkin R.M., Riddle D., Yousef A., Trojanowski J.Q. (2017). Novel conformation-selective alpha-synuclein antibodies raised against different in vitro fibril forms show distinct patterns of Lewy pathology in Parkinson’s disease. Neuropathol. Appl. Neurobiol..

[332] Vaikath N.N., Majbour N.K., Paleologou K.E., Ardah M.T., van Dam E., van de Berg W.D., Forrest S.L., Parkkinen L., Gai W.P., Hattori N. (2015). Generation and characterization of novel conformation-specific monoclonal antibodies for alpha-synuclein pathology. Neurobiol. Dis..

[333] Spencer B., Valera E., Rockenstein E., Overk C., Mante M., Adame A., Zago W., Seubert P., Barbour R., Schenk D. (2017). Anti-alpha-synuclein immunotherapy reduces alpha-synuclein propagation in the axon and degeneration in a combined viral vector and transgenic model of synucleinopathy. Acta Neuropathol. Commun..

[334] Sahin C., Lorenzen N., Lemminger L., Christiansen G., Moller I.M., Vesterager L.B., Pedersen L.O., Fog K., Kallunki P., Otzen D.E. (2017). Antibodies against the C-terminus of alpha-synuclein modulate its fibrillation. Biophys. Chem..

[335] Gupta V., Sudhakaran I.P., Islam Z., Vaikath N.N., Hmila I., Lukacsovich T., Kolatkar P.R., El-Agnaf O.M.A. (2020). Expression, purification and characterization of alpha-synuclein fibrillar specific scFv from inclusion bodies. PLoS ONE.

[336] Fassler M., Benaim C., George J. (2022). A Single Chain Fragment Variant Binding Misfolded Alpha-Synuclein Exhibits Neuroprotective and Antigen-Specific Anti-Inflammatory Properties. Cells.

[337] Kalsoom I., Wang Y., Li B., Wen H. (2023). Research Progress of alpha-Synuclein Aggregation Inhibitors for Potential Parkinson’s Disease Treatment. Mini Rev. Med. Chem..

[338] Jan A., Goncalves N.P., Vaegter C.B., Jensen P.H., Ferreira N. (2021). The Prion-Like Spreading of Alpha-Synuclein in Parkinson’s Disease: Update on Models and Hypotheses. Int. J. Mol. Sci..

[339] Hlushchuk I., Ruskoaho H., Domanskyi A., Airavaara M., Valimaki M.J. (2021). Domain-Independent Inhibition of CBP/p300 Attenuates Alpha-Synuclein Aggregation. ACS Chem. Neurosci..

[340] Tarutani A., Hasegawa M. (2019). Prion-like propagation of alpha-synuclein in neurodegenerative diseases. Prog. Mol. Biol. Transl. Sci..

[341] Pujols J., Pena-Diaz S., Lazaro D.F., Peccati F., Pinheiro F., Gonzalez D., Carija A., Navarro S., Conde-Gimenez M., Garcia J. (2018). Small molecule inhibits alpha-synuclein aggregation, disrupts amyloid fibrils, and prevents degeneration of dopaminergic neurons. Proc. Natl. Acad. Sci. USA.

[342] Pena-Diaz S., Pujols J., Vasili E., Pinheiro F., Santos J., Manglano-Artunedo Z., Outeiro T.F., Ventura S. (2022). The small aromatic compound SynuClean-D inhibits the aggregation and seeded polymerization of multiple alpha-synuclein strains. J. Biol. Chem..

[343] Staats R., Brotzakis Z.F., Chia S., Horne R.I., Vendruscolo M. (2023). Optimization of a small molecule inhibitor of secondary nucleation in alpha-synuclein aggregation. Front. Mol. Biosci..

[344] Zhang J., Liu S., Wu Y., Tang Z., Wu Y., Qi Y., Dong F., Wang Y. (2023). Enlarged Perivascular Space and Index for Diffusivity Along the Perivascular Space as Emerging Neuroimaging Biomarkers of Neurological Diseases. Cell. Mol. Neurobiol..

[345] Zhou C., Jiang X., Guan X., Guo T., Wu J., Wu H., Wu C., Chen J., Wen J., Tan S. (2024). Glymphatic system dysfunction and risk of clinical milestones in patients with Parkinson disease. Eur. J. Neurol..

[346] Lian X., Liu Z., Gan Z., Yan Q., Tong L., Qiu L., Liu Y., Chen J.F., Li Z. (2025). Targeting the glymphatic system to promote alpha-synuclein clearance: A novel therapeutic strategy for Parkinson’s disease. Neural Regen. Res..

[347] Ran L., Fang Y., Cheng C., He Y., Shao Z., Kong Y., Huang H., Xu S., Luo X., Wang W. (2025). Genome-wide and phenome-wide studies provided insights into brain glymphatic system function and its clinical associations. Sci. Adv..

[348] Padilla-Godinez F.J., Ruiz-Ortega L.I., Guerra-Crespo M. (2022). Nanomedicine in the Face of Parkinson’s Disease: From Drug Delivery Systems to Nanozymes. Cells.

[349] Lazaro D.F., Lee V.M. (2024). Navigating through the complexities of synucleinopathies: Insights into pathogenesis, heterogeneity, and future perspectives. Neuron.

[350] Li S., Liu Y., Lu S., Xu J., Liu X., Yang D., Yang Y., Hou L., Li N. (2025). A crazy trio in Parkinson’s disease: Metabolism alteration, alpha-synuclein aggregation, and oxidative stress. Mol. Cell. Biochem..

[351] Qu L., Tang Y., Wu J., Yun X., Lo H.H., Song L., Wang X., Wang H., Zhang R., Liu M. (2024). FBXL16: A new regulator of neuroinflammation and cognition in Alzheimer’s disease through the ubiquitination-dependent degradation of amyloid precursor protein. Biomark. Res..

[352] Mehmood A., Ali W., Din Z.U., Song S., Sohail M., Shah W., Guo J., Guo R.Y., Ilahi I., Shah S. (2021). Clustered regularly interspaced short palindromic repeats as an advanced treatment for Parkinson’s disease. Brain Behav..

[353] Safari F., Hatam G., Behbahani A.B., Rezaei V., Barekati-Mowahed M., Petramfar P., Khademi F. (2020). CRISPR System: A High-throughput Toolbox for Research and Treatment of Parkinson’s Disease. Cell. Mol. Neurobiol..

[354] Mansour H.M., El-Khatib A.S. (2023). Exploring Parkinson-associated kinases for CRISPR/Cas9-based gene editing: Beyond alpha-synuclein. Ageing Res. Rev..

[355] Wuensche T.E., Pereira P.M., Windhorst A.D., Bjerregaard-Andersen K., Sotty F., Kallunki P., Jensen A., Bang-Andersen B., van Dongen G., Beaino W. (2024). New prospects for (89)Zr-immuno-PET in brain applications—Alpha-synucleinopathies. Nucl. Med. Biol..

[356] Xiang J., Zhang Z., Wu S., Ye K. (2025). Positron emission tomography tracers for synucleinopathies. Mol. Neurodegener..

[357] Alfaidi M., Barker R.A., Kuan W.L. (2024). An update on immune-based alpha-synuclein trials in Parkinson’s disease. J. Neurol..

[358] Saadh M.J., Muhammad F.A., Singh A., Mustafa M.A., Al Zuhairi R.A.H., Ghildiyal P., Hashim G., Alsaikhan F., Khalilollah S., Akhavan-Sigari R. (2024). MicroRNAs Modulating Neuroinflammation in Parkinson’s disease. Inflammation.

[359] Mahboob A., Ali H., AlNaimi A., Yousef M., Rob M., Al-Muhannadi N.A., Senevirathne D.K.L., Chaari A. (2024). Immunotherapy for Parkinson’s Disease and Alzheimer’s Disease: A Promising Disease-Modifying Therapy. Cells.

[360] Khan I., Preeti K., Fernandes V., Khatri D.K., Singh S.B. (2022). Role of MicroRNAs, Aptamers in Neuroinflammation and Neurodegenerative Disorders. Cell. Mol. Neurobiol..

[361] Forsyth C.B., Shannon K.M., Kordower J.H., Voigt R.M., Shaikh M., Jaglin J.A., Estes J.D., Dodiya H.B., Keshavarzian A. (2011). Increased intestinal permeability correlates with sigmoid mucosa alpha-synuclein staining and endotoxin exposure markers in early Parkinson’s disease. PLoS ONE.

[362] Suresh S.B., Malireddi A., Abera M., Noor K., Ansar M., Boddeti S., Nath T.S. (2024). Gut Microbiome and Its Role in Parkinson’s Disease. Cureus.

[363] He S., Ru Q., Chen L., Xu G., Wu Y. (2024). Advances in animal models of Parkinson’s disease. Brain Res. Bull..

